# Cytomegalovirus m154 Hinders CD48 Cell-Surface Expression and Promotes Viral Escape from Host Natural Killer Cell Control

**DOI:** 10.1371/journal.ppat.1004000

**Published:** 2014-03-13

**Authors:** Angela Zarama, Natàlia Pérez-Carmona, Domènec Farré, Adriana Tomic, Eva Maria Borst, Martin Messerle, Stipan Jonjic, Pablo Engel, Ana Angulo

**Affiliations:** 1 Institut d'Investigacions Biomèdiques August Pi i Sunyer, Barcelona, Spain; 2 Department of Histology and Embryology, Faculty of Medicine, University of Rijeka, Rijeka, Croatia; 3 Department of Virology, Hannover Medical School, Hannover, Germany; 4 Immunology Unit, Department of Cell Biology, Immunology, and Neurosciences, Medical School, University of Barcelona, Barcelona, Spain; La Jolla Institute for Allergy and Immunology, United States of America

## Abstract

Receptors of the signalling lymphocyte-activation molecules (SLAM) family are involved in the functional regulation of a variety of immune cells upon engagement through homotypic or heterotypic interactions amongst them. Here we show that murine cytomegalovirus (MCMV) dampens the surface expression of several SLAM receptors during the course of the infection of macrophages. By screening a panel of MCMV deletion mutants, we identified m154 as an immunoevasin that effectively reduces the cell-surface expression of the SLAM family member CD48, a high-affinity ligand for natural killer (NK) and cytotoxic T cell receptor CD244. m154 is a mucin-like protein, expressed with early kinetics, which can be found at the cell surface of the infected cell. During infection, m154 leads to proteolytic degradation of CD48. This viral protein interferes with the NK cell cytotoxicity triggered by MCMV-infected macrophages. In addition, we demonstrate that an MCMV mutant virus lacking m154 expression results in an attenuated phenotype *in vivo*, which can be substantially restored after NK cell depletion in mice. This is the first description of a viral gene capable of downregulating CD48. Our novel findings define m154 as an important player in MCMV innate immune regulation.

## Author Summary

Cytomegalovirus (CMV) has developed diverse tactics to elude the host immune response and guarantee its survival. The signalling lymphocyte-activation molecules (SLAM) family of receptors encompasses a number of adhesion molecules expressed on the surface of leukocytes that play critical roles in both innate and adaptive immunity. In this study, we report that murine CMV drastically reduces the expression of several SLAM family receptors at the cell surface of infected macrophages, most likely as part of its immunoevasion mechanisms. We have identified a murine CMV gene product (m154) that downregulates CD48, a SLAM family member that functions as a ligand of CD244, a molecule involved in the regulation of natural killer (NK) and cytotoxic T cell functions. We show that during infection, m154 targets CD48 for degradation. Moreover, this viral protein contributes to increased MCMV growth during acute infection in the mouse by protecting against NK cell mediated surveillance. These findings are important for better understanding CMV pathogenesis, and provide a novel example of host innate immune subversion by CMV.

## Introduction

Pathogens have recourse to innumerable tactics for evading host immune surveillance. Viruses, and in particular large DNA viruses such as herpesviruses, are endowed with the capacity to encode multiple products committed to altering, during all stages of their life cycle, several functions of the innate and adaptive immune system. The homeostatic equilibrium achieved between host immune responses and viral immune escape mechanisms empowers these viruses to successfully establish their characteristic lifelong infections. Human cytomegalovirus (CMV), the prototype β-herpesvirus, usually leads to asymptomatic infections in healthy individuals where it remains in a latent state for life, going through sporadic reactivation and leading to severe diseases in immunocompromised patients [1], [2]. The generation of an efficient host-elicited immune response against CMV includes the induction of natural killer (NK) cells, antibody and T-cell mediated responses [3]. As a consequence, CMV has evolved diverse countermeasures to avoid recognition by T cells, allowing it to interfere with the surface expression of major histocompatibility complex class I (MHC class I) and class II and costimulatory molecules, compromising antigen presentation [3]–[6]. Likewise, the virus counteracts NK cell triggering, primarily by suppressing the expression of ligands for activating receptors while preserving engaged inhibitory receptors [7]–[9]. In addition, CMV alters the function of cytokines and their receptors, and interacts with complement factors. While great strides have been made in recent years in identifying CMV inhibitors of immune response mechanisms, current consensus is that among the vast amount of genetic CMV material still requiring a functional assignment, the virus harbours as yet uncovered immunoevasins directed against already known or new immunological targets. Due to the species-specific nature of human CMV (HCMV) replication, infection of mice with murine CMV (MCMV) has proven to be an invaluable model for studying aspects of the biology underlying CMV infection. In this regard, the MCMV system has been widely used to unveil new immunomodulatory molecules and to explore their roles in infection and viral pathogenesis [10].

The signalling lymphocyte-activation molecules (SLAM) family of cell-surface receptors is a distinct structural subgroup of the immunoglobulin (Ig) superfamily differentially expressed on hematopoietic cells and found to play pivotal roles in both innate and adaptive immunity [11]–[13]. Among other activities, SLAM immunomodulatory receptors regulate cell adhesion, cytokine production, and cytotoxicity of NK and CD8^+^ T cells. The SLAM family currently consists of nine members, CD48, CD84, CD150 (SLAM), CD229, CD244 (2B4), CD319 (CRACC), CD352 (SLAMF6, NTB-A; Ly108), CD353 (BLAME) and SLAMF9. One of the hallmarks of this class of receptors is that they interact with members of the same family via their amino-terminal Ig-V domains. While most of them are typically self-ligands, participating in homophilic interactions, CD48 is a heterophilic receptor for CD244 [14]. The cytoplasmic domain of most SLAM family members carry one or more copies of a distinctive immunoreceptor intracellular tyrosine-based switch motif (ITSM) [13], [15]. Upon receptor engagement, these motifs undergo phosphorylation and recruit with high affinity and specificity adaptor molecules like the SLAM-associated protein (SAP) [11], [16].

In particular, CD48 is a GPI-anchored glycoprotein with expression in a broad range of cells of the hematopoietic lineage, especially on antigen-presenting cells [17]. CD244, the high-affinity counter receptor of CD48 both in humans and mice, is a transmembrane surface glycoprotein with an intracellular tail containing four ITSMs. It is highly expressed on NK cells, and to a lesser extent on other cytotoxic cells such as CD8^+^ T cells, basophils, and eosinophils. CD244 is an important activating receptor for the regulation of CD8^+^ T and mature NK cells, promoting cell-mediated cytotoxicity and cytokine release [18]–[20]. Engagement of CD244 by its ligand leads to the polarization and release of cytolytic granules into the contact zone between NK and target cells [21].

SLAM family receptors have been shown to play specific roles in viral pathogenesis. Various morbilliviruses, including the highly contagious measles virus, employ CD150 as the principal receptor to enter into a subset of immune cells, facilitating their spread and contributing to viral-induced immunosuppression [22], [23]. In response to Epstein-Barr virus infection, CD48 is strongly induced on the surface of B lymphocytes and may aid viral trafficking [24]. In addition, we have recently shown that HCMV encodes UL7, a CD229 structural homologue capable of interfering with proinflammatory responses [25]. The role of SLAM receptors in antiviral immunity has been clearly documented in the X-linked lymphoproliferative syndrome, a rare immunodeficiency human disease in which impaired signalling functions of the SLAM receptors, stemming from mutations in the SAP-encoding gene, is associated with an extreme sensitivity to infection with Epstein-Barr virus [26]. Therefore, since SLAM receptors are active components of host immunity, viruses might have evolved immune evasion manoeuvres to specifically ablate triggering of such receptors. Indeed, this is the case for HIV-1, which utilizes Vpu to elude NK cell recognition through the downregulation of NTB-A expression on the surface of infected CD4^+^ T cells [27]. Whether this is a more generalized phenomenon and what consequences modulating SLAM receptors may cause in the infected host remain unknown.

In this study we show that MCMV infection efficiently decreases the expression of several SLAM family receptors at the cell surface of macrophages, and we pinpoint m154 as the viral downregulator of CD48. We found that m154 helps to debilitate the effectiveness of anti-MCMV triggered NK cell responses, thereby meliorating viral growth *in vivo*. Thus, we present here a novel strategy evolved by CMV to subvert detection by NK cells during acute infection, based on the modulation of a SLAM family member.

## Results

### Expression of SLAM receptors on the surface of mouse peritoneal macrophages

**Figure 1:**
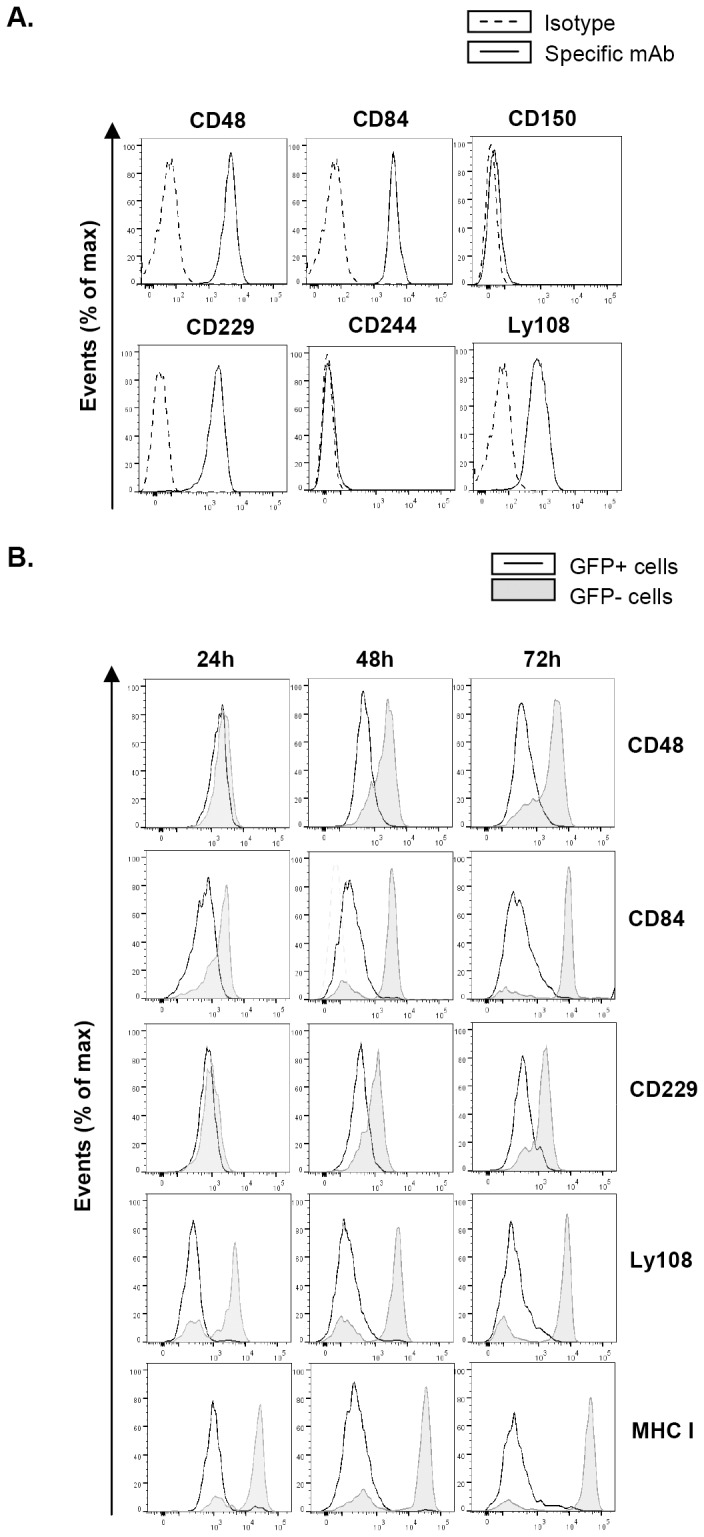
MCMV infection leads to reduced expression of SLAM receptors on the macrophage surface. (A) Surface expression of the SLAM receptors CD48, CD84, CD150, CD229, CD244, and Ly108 on peritoneal macrophages was tested by flow cytometry using specific mAbs against each of these molecules (black line histograms). Isotype IgGs directly conjugated with PE or APC were used as negative controls (dashed line histograms). A representative experiment out of three performed is shown. (B) Peritoneal macrophages were mock-infected or infected with MCMV-GFP at an moi of 2 and analyzed by flow cytometry at 24, 48 and 72 hours after infection for surface expression of CD48, CD84, CD229, Ly108, and MHC class I (MHC I) as indicated in A. Open histograms represent the expression of these molecules on MCMV-infected (GFP-positive) cells, and shaded histograms represent the expression on uninfected (GFP-negative) cells from the same culture.

SLAM family members are differentially expressed among hematopoietic cells. As macrophages play a key role in MCMV infection with regard to viral replication, dissemination, and the establishment of latency [28]–[30], and constitute one of the principal effectors of innate immunity, we selected this particular cell type to explore potential SLAM perturbations upon MCMV infection. Using flow cytometry, we first assessed whether CD48, CD84, CD150, CD229, CD244 and Ly108 were present on the surface of thioglycollate-elicited peritoneal macrophages. The lack of a commercially available antibody against CD319, CD353, and SLAMF9 prevented the study of these receptors in this cell type. As shown in Figure 1A, the SLAM receptors CD48, CD84, CD229, and Ly108 were expressed on the macrophage surface, whereas CD150 and CD244 could not be detected. We found however, that CD150 was present at the surface of LPS-treated mouse peritoneal macrophages (data not shown), consistent with earlier studies [31]. Thus, peritoneal macrophages represent an MCMV permissive cell type expressing a number of SLAM receptors, allowing us to examine whether members of this family could be targets for modulation during MCMV infection.

### Substantial reduction of cell-surface expression of SLAM receptors during the course of MCMV infection

We then infected peritoneal macrophages with MCMV-GFP at a multiplicity of infection (moi) of 2. The use of MCMV-GFP, based on the bacterial artificial chromosome (BAC)-cloned MCMV genome pSM3fr-GFP, which contains a GFP gene inserted within the *ie2* locus [32], allowed us to track and selectively analyze infected cells in the cultures. Under these conditions, infection rates reached approximately 50%. At different times (24 h, 48 h, and 72 h) after infection, cells were stained for the surface expression of CD48, CD84, CD229, and Ly108. Notably, MCMV infection resulted in the significant progressive downregulation of all the four receptors analyzed over the course of the infection, when compared to both non-infected cells (GFP negative) from the same culture (Figure 1B) or with mock-infected macrophages (data not shown). Surface reductions in CD84 and Ly108 were already observed at 24 h post-infection (hpi), and at 48 hpi for CD48 and CD229, becoming for all four receptors more pronounced at 72 hpi. Thus, by 72 hpi macrophages demonstrated a dramatic loss in expression of the four SLAM receptors analyzed. As expected [6], a significant surface decrease in MHC class I molecules was also detected in infected cells. Similar results were obtained when experiments were performed with wild-type (wt) MCMV not expressing GFP (data not shown).

**Figure 2:**
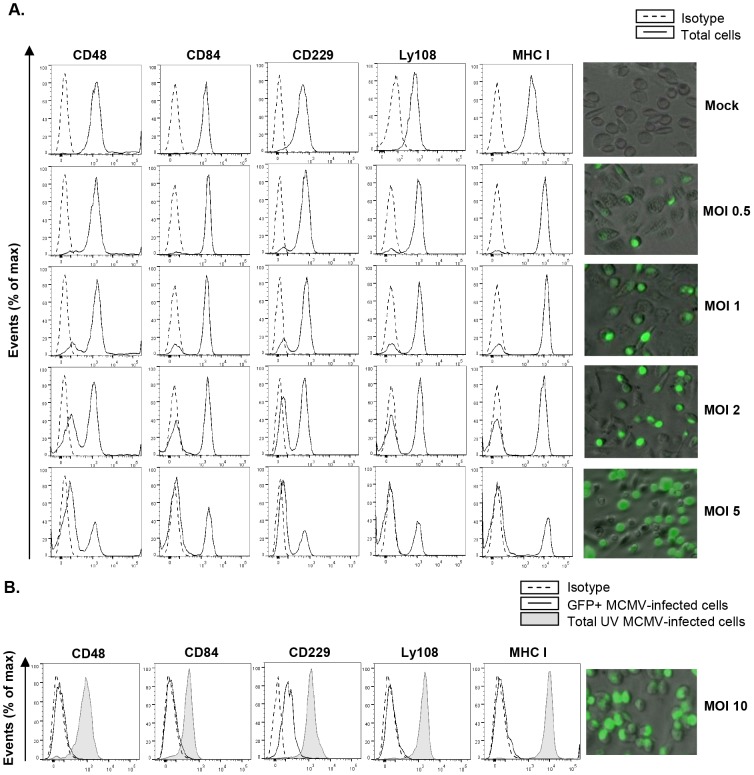
MCMV-induced downmodulation of SLAM receptors correlates with the extent of infection and depends on viral gene expression. (A) Peritoneal macrophages were mock-infected or infected for 72 h with MCMV-GFP at the different moi indicated, and analyzed by flow cytometry for surface expression of CD48, CD84, CD229, Ly108 and MHC class I (MHC I) as described in Figure 1. Black line histograms represent the expression of these molecules on the total number of cells alive in the cultures (including both MCMV-infected GFP-positive cells and uninfected GFP-negative cells), and dashed line histograms represent isotype controls. Micrographs of the corresponding infections are shown in the right panels. (B) Same as in A, except that an moi of 10 was used, and macrophages were also exposed for 72 h to the same amount of MCMV-GFP UV-inactivated. Open histograms represent the expression of these molecules on MCMV-infected cells from the MCMV-GFP treated cultures and shaded histograms represent the total number of cells alive in the MCMV-GFP UV-inactivated exposed cultures. Isotype IgGs were used as negative controls (dashed line histograms). A micrograph of the MCMV-GFP infection at an moi of 10 (GFP-positive, ∼95% in the culture) is shown on the right panel. A representative experiment out of two performed is shown.

We further analyzed the effect of the viral dose on the alteration of SLAM surface expression by infecting peritoneal macrophages at different mois, ranging from 0.5 (∼5% infected macrophages) to 5 (∼70% infected macrophages), with MCMV-GFP. As depicted in Figure 2A, there was a strong dependency on the viral dose for cell-surface reduction of SLAM receptor expression concomitant with the downmodulation of MHC class I, which in turn correlated with the extent of infected peritoneal macrophages.

To determine whether viral gene expression was required for SLAM downregulation, macrophages were treated with UV-inactivated MCMV. The results showed no decrease in CD48, CD84, CD229, or Ly108 surface expression after infection of macrophages for 72 h with the UV-inactivated virus (Figure 2B), indicating that SLAM downregulation could be attributed to specific MCMV genome-encoded products. Moreover, for Ly108, cell-membrane expression levels after infection with UV-inactivated MCMV were even higher than those of uninfected cells, most likely due to the viral-dependent macrophage activation (data not shown).

Altogether these results show that MCMV encodes gene products that efficiently diminish the cell-surface levels of SLAM family members.

### Identification of *m154* as the MCMV gene that interferes with CD48 cell-surface expression

Since CD244, the high affinity receptor for CD48, is expressed in NK and cytotoxic CD8^+^ T cells known to play a prominent role in the clearance of MCMV infection, we decided to further explore the consequences of the cell-surface depletion of CD48, and sought to identify the viral product(s) causing it.

**Figure 3:**
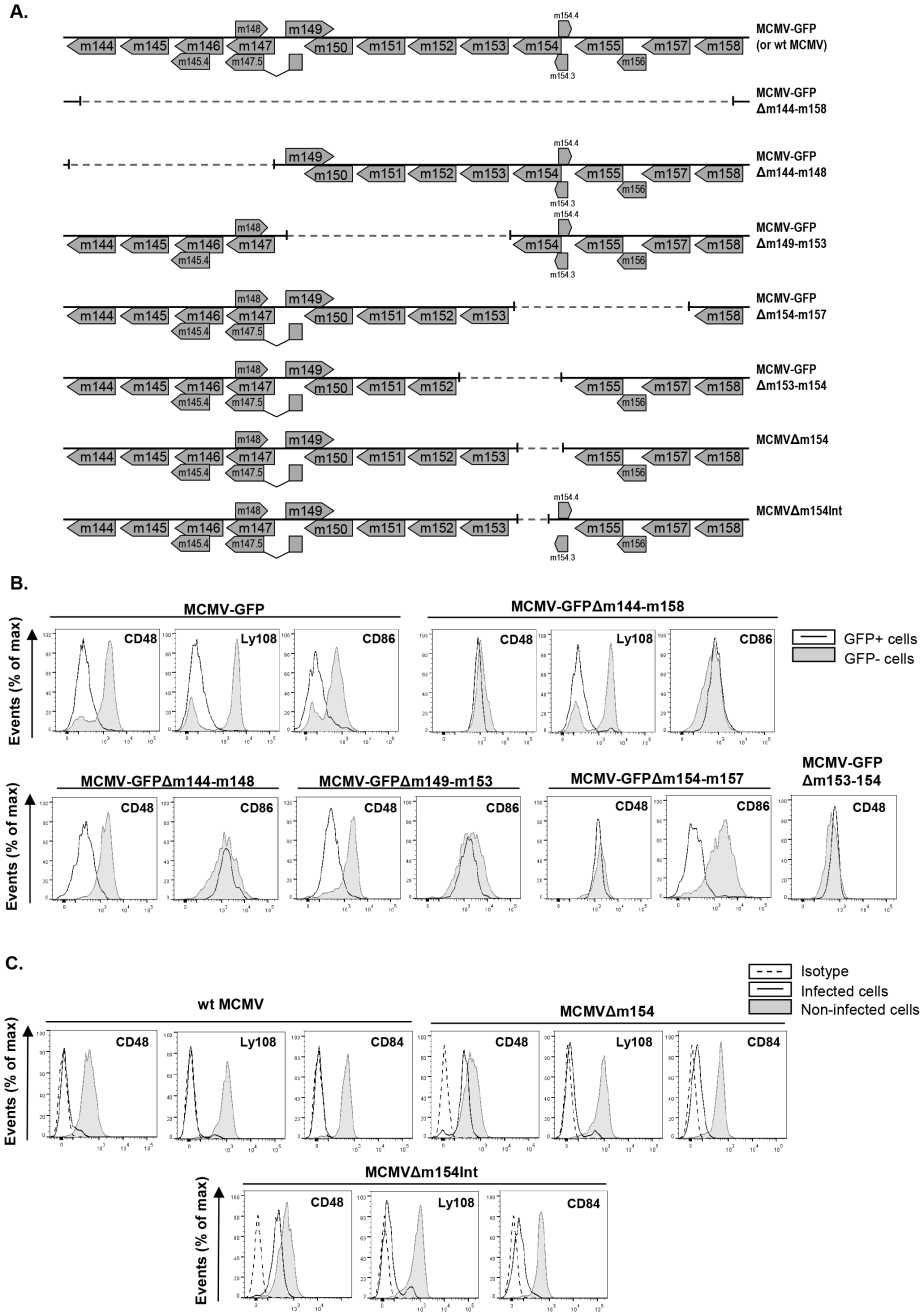
Deletion of *m154* results in decreased CD48 surface density on MCMV-infected macrophages. (A) Schematic depiction of the region between the *m144* and *m158* genes in parental MCMV-GFP or wt MCMV, MCMV-GFPΔm144-m158, MCMV-GFPΔm144-m148, MCMV-GFPΔm149-m153, MCMV-GFPΔm154-m157, MCMV-GFPΔm153-m154, MCMVΔm154, and MCMVΔm154Int used in the analysis. (B) Peritoneal macrophages were mock-infected or infected for 72 h with MCMV-GFP, MCMV-GFPΔm144-m158 MCMV-GFPΔm144-m148, MCMV-GFPΔm149-m153, MCMV-GFPΔm154-m157, and MCMV-GFPΔm153-m154 at an moi of 2 and analyzed by flow cytometry for surface expression of CD48, Ly108, or CD86 as described in Figure 1. Open histograms represent the expression of these molecules on infected (GFP-positive) cells and shaded histograms represent the expression on uninfected (GFP-negative) cells from the same cultures. A representative experiment out of at least two performed is shown. (C) Same as in B, except that infections were performed with wt MCMV, MCMVΔm154, MCMVΔm154Int at an moi of 10 and analyzed by flow cytometry for surface expression of CD48, Ly108, and CD84. Open histograms represent the expression of these molecules on infected cultures and shaded histograms represent the expression on uninfected cultures. Isotype IgGs were used as negative controls (dashed line histograms). A representative experiment out of three performed is shown.

The potential modulators of SLAM receptors would most likely be genes dispensable for viral replication *in vitro*. Thus, to identify the MCMV gene product(s) that might mediate the downregulation of CD48, we systematically screened the viral genome utilizing a panel of mutant viruses bearing deletions of approximately 10–15 kbp each in non-essential regions. These mutant viruses were based on the BAC-cloned MCMV genome containing the GFP. Peritoneal mouse macrophages were infected with wild-type and mutant MCMVs, and at 72 hpi were tested for surface expression of CD48. After infection with the deletion mutant MCMV-GFPΔm144-m158 (Figure 3A) missing genes extending from *m144* to *m158*, cell-surface CD48 was restored, reaching levels comparable to that of non-infected cells (Figure 3B). As expected, due to the lack of the *m147.5* gene this deletion mutant was also capable to revert the cell-surface expression of CD86 [33], whereas it did not significantly affect the downregulation of other SLAM receptors, such as Ly108. At this point, three additional viral mutants, MCMV-GFPΔm144-m148, MCMV-GFPΔm149-m153, and MCMV-GFPΔm154-m157 all containing smaller specific deletions within the *m144-m157* region (from *m144* to *m148*, from *m149* to *m153*, and from *m154* to *m157*, respectively) (Figure 3A) were assessed for their capability to interfere with CD48. As shown in Figure 3B, only the MCMV mutant in which the genetic region encompassing *m154* to *m157* was removed, efficiently relieved CD48 downregulation, while levels of CD86 remained similar to those present in wt MCMV-infected macrophages. CD86, however, was not reduced from the macrophage surface after infection with either MCMV-GFPΔm144-m148 or MCMV-GFPΔm149-m153, mutants that do lack the *m147.5* gene. To further narrow down the possible viral CD48 downregulators, we examined two additional viral mutants containing deletions within the *m153*-*m157* genomic region, MCMV-GFPΔm153-m154 and MCMV-GFPΔm155-m157 (Figure 3A and data not shown). Notably, the MCMV mutant lacking *m153* and *m154* genes, but not the viral mutant missing genes *m155* to *m157*, reverted CD48 downregulation (Figure 3B, and data not shown). As a role of the *m153* gene in CD48 cell-surface alteration had been excluded after analyzing MCMV-GFPΔm149-m153, we deduced that the *m154* gene product was the one leading to reduced macrophage-surface expression of CD48 during MCMV infection. This observation was confirmed with a viral mutant bearing a deletion in *m154*, MCMVΔm154 (Figure 3A), which was able to ablate downregulation of CD48 to an extent comparable to that of mock-infected cells, whereas it maintained the downregulation of Ly108 and CD84 (Figure 3C). As Tang and co-workers [34] in a reassessment of global MCMV ORFs using DNA microarray analysis reported two additional small ORFs, m154.3 and m154.4, potentially expressed in infected NIH 3T3 cells, that partially overlapped with ORF m154 and which therefore were interrupted in the deletion mutant MCMVΔm154, a new recombinant MCMV carrying a smaller internal deletion in *m154* that preserved intact both *m154.*3 and *m154.4* (MCMVΔm154Int) was generated. In a manner similar to MCMVΔm154, MCMVΔm154Int did not significantly alter CD48 surface levels (Figure 3C). These data further confirmed that the observed rescue of CD48 surface density in infected macrophages was the result of deleting the *m154* gene. Thus, we concluded that m154 abrogates the surface expression of CD48.

### Characterization of the m154 gene product

**Figure 4:**
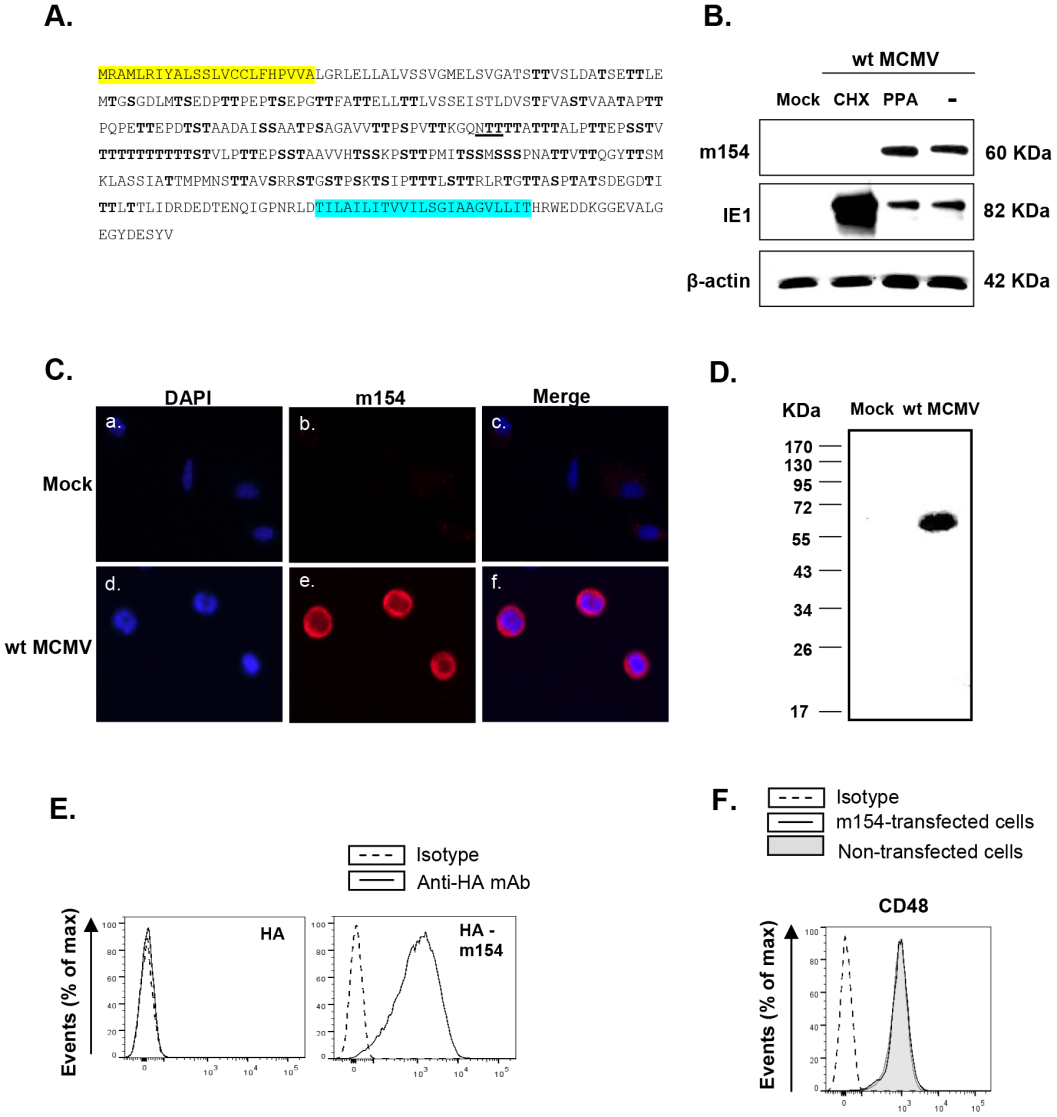
Analysis of MCMV m154. (A) The deduced amino acid sequence of m154 is shown, with the predicted leader peptide in yellow and the transmembrane region in blue. The putative N-linked glycosylation site (underlined) and O-glycosylation sites (bold) are indicated. (B) Peritoneal macrophages were mock-infected (mock) or infected with wt MCMV at an moi of 10 in the absence (lane -) or presence of cycloheximide and actinomycin D (lane CHX), or phosphonoacetic acid (lane PPA). Cell lysates were prepared at 72 hpi, except for the cycloheximide treated samples that were collected 16 hpi, separated under reducing conditions by SDS-PAGE (10%), and transferred to a nitrocellulose membrane. The blot was proved with the anti-m154 mAb or anti-MCMV IE1 (Croma 101) mAb followed by anti-mouse IgG HRP. A mAb anti-β-actin followed by an anti-rabbit IgG HRP was used as an internal control. (C) Peritoneal macrophages, either mock-infected or infected with wt MCMV at an moi of 10 for 72 h, were fixed, permeabilized, and stained with anti-m154 mAb followed by an anti-mouse IgG Alexa fluor 555. Nuclei were stained with the DAPI reagent. The cells were examined under a microscope at 405 nm (DAPI, panels a and d) and at 555–565 nm (Alexa-555, panels b and e). The overlaid images are shown in panels c and f. (×10 magnification). (D) Peritoneal macrophages, either mock-infected (mock) or infected with wt MCMV at an moi of 10, were surface-labeled with biotin. Cell lysates were prepared at 72 hpi and subjected to immunoprecipitation with anti-m154 mAb, followed by SDS-PAGE (10%) separation and Western blot analysis as indicated in B, using streptavidin-POD conjugate. Molecular weights in kilodaltons are indicated. (E) Flow cytometry analysis of 300.19 cells stably transfected with empty pDisplay vector expressing HA (left panel) or the construct HA-m154 (right panel) and stained after 24 h with biotin anti-HA mAb (black line) or isotype control (dashed line) followed by streptavidin-PE. (F) 300.19 cells non-transfected (shaded histogram) or stably transfected with the construct HA-m154 (black line histogram) were analyzed by flow cytometry for surface expression of CD48 as indicated in Figure 1. The corresponding isotype was used as a negative control (dashed line histogram).

The *m154* gene belongs to the *m145* gene family [35], comprised of eleven members, some of which encode molecules that adopt an MHC class I fold [36] and which are known to be involved in the modulation of immune responses. In contrast to other members of this family, the *m154* gene has no homology with MHC class I genes. It encodes a 368-aa type I transmembrane protein with a 23-aa putative N-terminal signal peptide, a 300-aa ectodomain, a 23-aa transmembrane domain, and a 22-aa C-terminal cytoplasmic tail (Figure 4A). The ectodomain is a mucin-like domain displaying a striking number of serine (29) and threonine (84) residues that are potential O-linked glycosylation sites, and contains one putative N-glycosylation site (at position 161). A search of the available sequence databases using the m154 deduced amino acid sequence revealed no significant degree of sequence identity between m154 and other known viral or host proteins.

In order to examine m154 expression during the viral infection, we raised a specific monoclonal antibody (mAb; m154.4.113) against the protein, using a peptide corresponding to its cytoplasmic tail as an immunogen. Peritoneal macrophages, either mock-infected or infected for 72 h with wt MCMV, were analyzed by Western blot with this mAb. A single protein band with an apparent molecular mass of ∼60 kDa was detected only in the infected cell (Figure 4B). The migration of the detected protein differed from the predicted size of the mature m154, which is 38 kDa, being highly suggestive of an extensive glycosylation occurring via its copious serine and threonine residues. To identify the expression kinetic class of m154, we infected macrophages in the presence of either the viral DNA synthesis inhibitor phosphonoacetic acid (PPA), which prevents late viral gene expression, or the protein translation inhibitor cycloheximide (CHX), which selectively limits viral gene expression to immediate early genes. As shown in Figure 4B, m154 was not recovered after release from the CHX block in the presence of actinomycin D, whereas under these conditions, the major immediate early MCMV protein IE1 was abundantly found, as expected. m154, however, was readily detected after PPA treatment, indicating that this viral protein is expressed with early kinetics.

Infected macrophages were also examined by indirect immunofluorescence to determine the subcellular localization of m154. The protein was strongly expressed on the cell membrane and to a lesser extent in the cytoplasm (Figure 4C). Biotin-labelling of proteins on the surface of wt MCMV-infected macrophages, followed by immunoprecipitation with the anti-m154 mAb, SDS-PAGE, and subsequent Western blot probed with labelled streptavidin, confirmed the presence of m154 at the cell surface (Figure 4D). Localization of m154 on the cell surface was also observed after ectopic expression of *m154*. Thus, when 300.19 cells stably transfected with an HA-m154 fusion protein containing the influenza hemagglutinin (HA) epitope tag inserted at the N-terminal end of m154 were analyzed by flow cytometry using an anti-HA antibody, a cell-surface pattern of HA staining was observed (Figure 4E). It must be noted, however, that when expressed in isolation, m154 exerted no overt effects on the surface levels of CD48, which was constitutively expressed in 300.19 cells (Figure 4F). This result suggests the need of additional MCMV encoded proteins or virally induced cellular molecules for m154 to operate appropriately.

**Figure 5:**
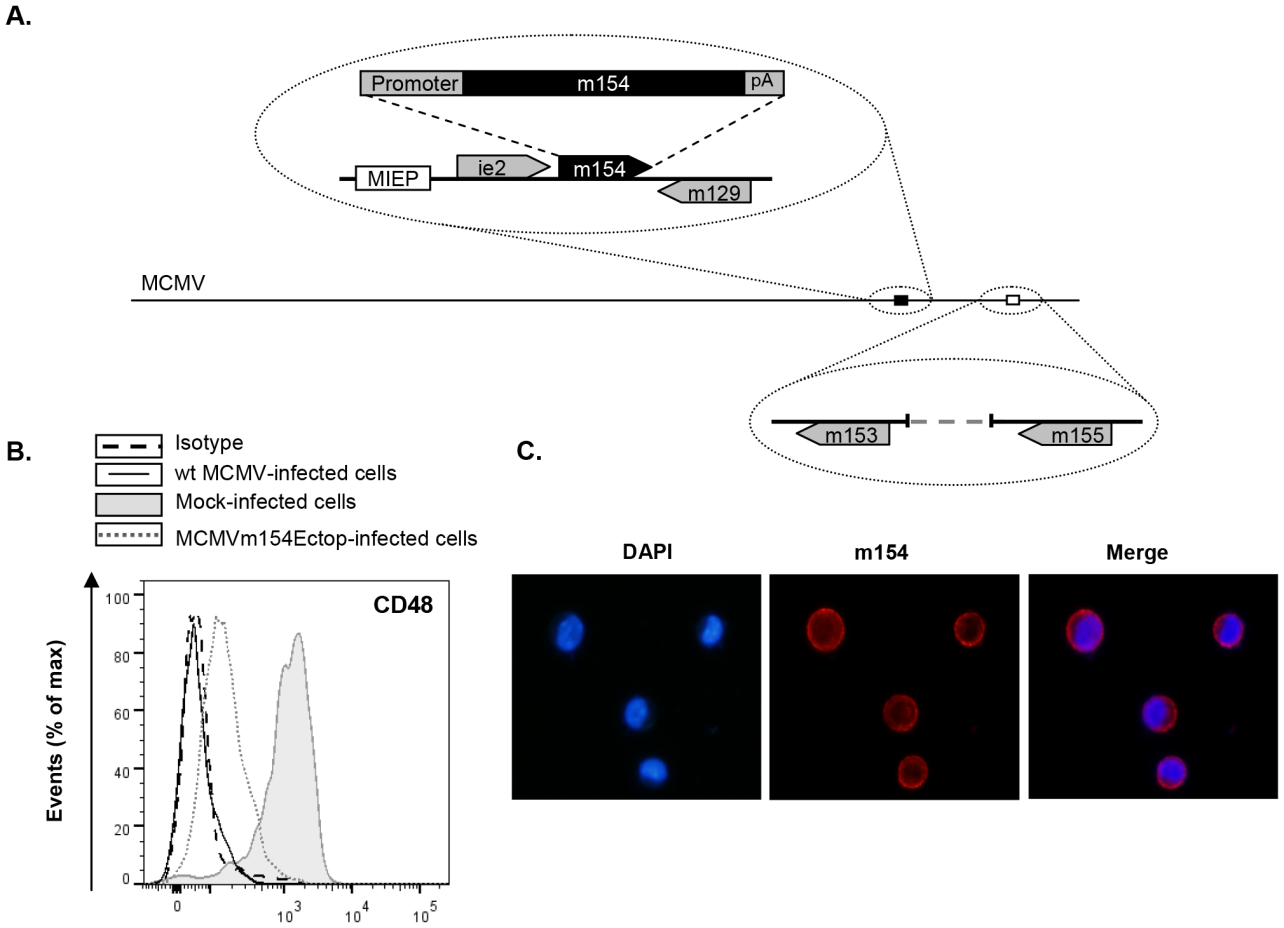
The *m154* gene ectopically expressed within the MCMVΔm154 genome decreases CD48 surface levels on MCMV-infected macrophages. (A) Schematic depiction of MCMVm154Ectop, with an expansion of the *m153-m155* region (represented by an open box) to show the deletion of the *m154* gene, and of part of the MIE region (represented by a black box) to indicate the location of the *m154* gene inserted (together with 210 nt of its corresponding putative promoter and 60 nt including its putative polyA signal [pA]) in between the *ie2* and the *m129* genes. The major IE promoter (MIEP) is marked. (B) Peritoneal macrophages were mock-infected or infected for 72 h with wt MCMV or MCMVm154Ectop at an moi of 10 and analyzed by flow cytometry for surface expression of CD48 as described in Figure 1. Shaded histograms represent CD48 expression on mock-infected cells, and black line and dotted-line histograms represent CD48 expression on cells from wt MCMV- and MCMVm154Ectop-infected macrophages, respectively. Isotype IgG was used as a negative control (dashed line histograms). A representative experiment out of two performed is shown. (C) Peritoneal macrophages, infected with MCMVm154Ectop at an moi of 10 for 72 h, were fixed, permeabilized, and stained with anti-m154 mAb followed by an anti-mouse IgG Alexa fluor 555, and analyzed as indicated in Figure 4C. Shown are nuclei stained with the DAPI reagent (left panel), cultures stained for m154 (middle panel), and the overlaid image (right panel). Images were captured at 40× magnification.

To asses the ability of m154 to downregulate CD48 when ectopically expressed in the context of an MCMV infection, we generated a viral mutant (MCMVm154Ectop, Figure 5A) in which we inserted the *m154* ORF plus 210 nt of its corresponding putative promoter and 60 nt including its putative polyA signal, into the genome of MCMVΔm154 behind the *ie2* ORF. As shown in Figure 5B, MCMVm154Ectop largely reduced CD48 macrophage-surface levels when examined at 72 hpi, time at which m154 could be clearly detected by indirect immunofluorescence in the MCMVm154Ectop-infected cell, where it displayed a distribution comparable to that observed during wt MCMV infection (compare Figure 5C and Figure 4C).

Based on all these findings, we concluded that m154, the MCMV downregulator of CD48, is an early-phase mucin-like protein with a predominant cell-surface localization in the infected cell.

### m154 downregulates CD48 via a degradation route

**Figure 6:**
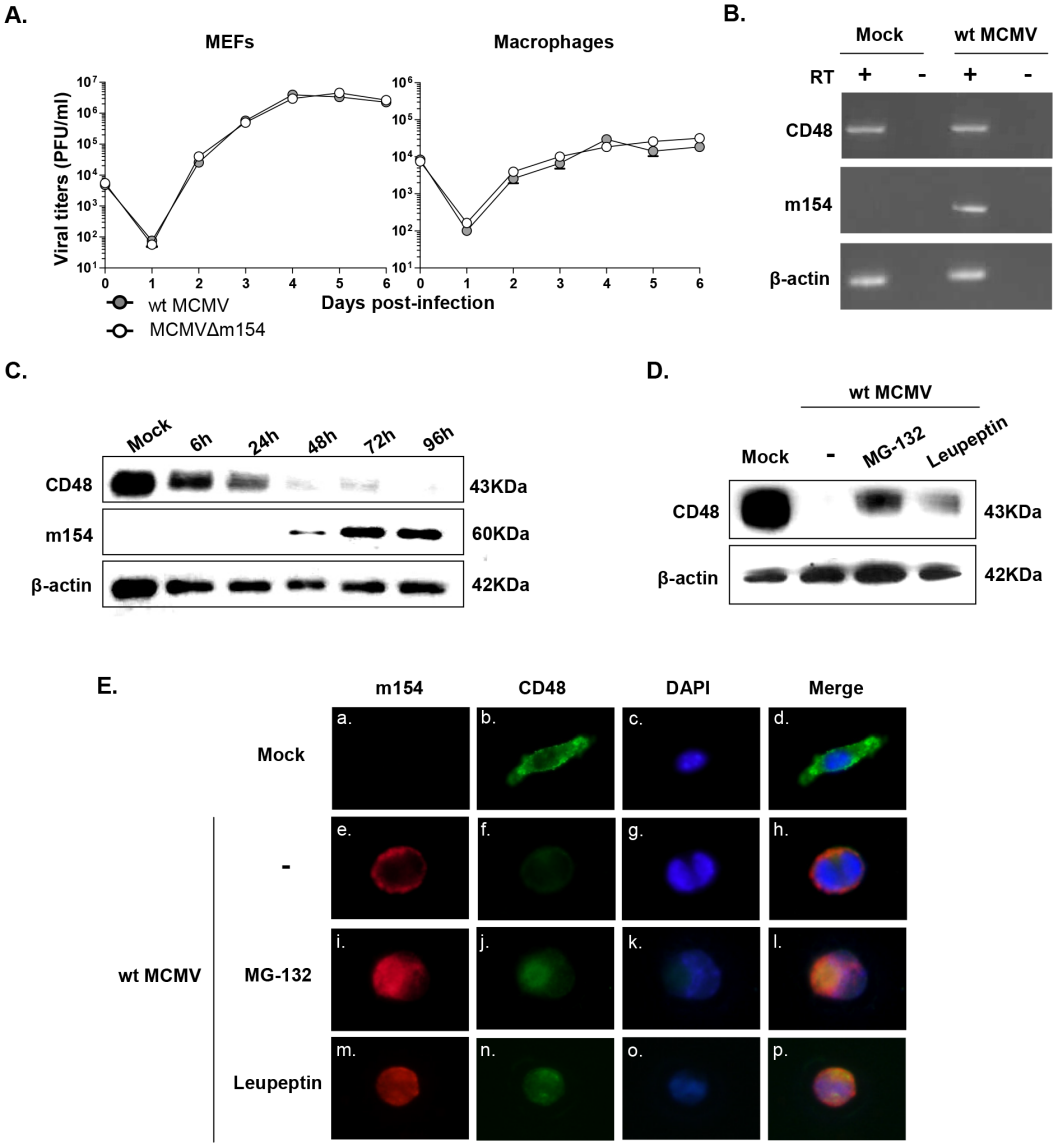
CD48 undergoes proteolytic degradation during MCMV infection. (A) MEFs and peritoneal macrophages were infected at an moi of 0.025 or 0.1, respectively with wt MCMV and MCMVΔm154. At the indicated days after infection the amount of extracellular (MEFs) or cell-associated (macrophages) infectious virus present in the cultures was determined. Each data point represents the average and standard deviation of three separate cultures. (B) Peritoneal macrophages were mock-infected or infected with wt MCMV at an moi of 10 for 72 h. Whole-cell RNA was harvested, treated with DNase and reverse-transcribed using oligo(dT). PCRs were performed using primer sets specific for murine CD48, m154, and β-actin. The amplified products were separated on agarose gels and visualized with ethidium bromide staining. As shown, specific PCR-amplified products were not detected in control reactions in which the reverse transcriptase (RT) was not added during the reverse transcription reaction. (C) Peritoneal macrophages were mock-infected or infected with wt MCMV at an moi of 10. At the indicated time points after infection, cell lysates were prepared, and subjected to Western blot analysis as indicated in Figure 4B using an anti-mouse CD48 mAb or the anti-m154 mAb followed by anti-Armenian hamster HRP or by anti-mouse IgG HRP, respectively. A mAb anti-β-actin followed by anti-rabbit IgG HRP was used as an internal control. (D) Peritoneal macrophages were mock-infected (mock) or infected with wt MCMV at an moi of 10 in the absence (-) or presence of MG-132 or leupeptin. Cell lysates were prepared at 72 hpi and subjected to Western blot analysis as indicated in C. (E) Peritoneal macrophages were mock-infected or infected with wt MCMV at an moi of 10 for 72 h. When indicated, cultures were treated with MG-132 or leupeptin. Cells were fixed, permeabilized, and stained with anti-CD48-Alexa Fluor 488, and anti-m154 mAb followed by an anti-mouse IgG Alexa fluor 555, and analyzed as indicated in Figure 4C. Shown are representative cells from cultures stained for m154 (panels a, e, i, and m), for CD48 (panels b, f, j, and n), nuclei stained with the DAPI reagent (panels c, g, k, and o), and overlaid images (panels d, h, l, and p). Images were captured at 40× magnification.

Although MCMV-encoded products with immunomodulatory properties are not believed to play a role in the viral replication cycle, we analyzed whether m154 affected MCMV growth in tissue culture. To this end, single-step growth curves of MCMVΔm154 and wt viruses were determined in mouse embryo fibroblasts (MEFs) and peritoneal macrophages after infection at a low moi. MCMVΔm154 displayed plaque morphologies in MEFs and growth kinetics in both cell types that were indistinguishable from those of wt virus, confirming the lack of involvement of m154 in the viral replication cycle (Figure 6A and data not shown).

In addressing the mechanism by which m154 downregulates CD48, we first considered the possibility that this viral protein may affect CD48 transcription. However, when we compared CD48 RNA levels in wt MCMV-infected macrophages with mock-infected cells by reverse transcriptase (RT)-PCR, we found no substantial change in CD48 mRNA content (Figure 6B). This observation suggested that CD48 expression was being altered through post-transcriptional mechanisms. Therefore, we examined CD48 protein in cell lysates of wt MCMV-infected cells at different time points by using Western blot. As depicted in Figure 6C, CD48 (∼40 kDa band) levels were drastically lower in total cell lysates of infected macrophages, especially after 48 h of infection. This decrease in CD48 occurred concomitantly with the appearance of m154, which was readily detected 48 h after infection, reaching a maximum at 72 hpi and then continuing to accumulate in the infected cell. Thus, the data pointed towards proteolytic degradation of CD48 during MCMV infection. To assess which protein degradation pathway was involved, we used proteasomal or lysosomal proteolysis inhibitors. MG-132 is considered a specific 26S proteasome inhibitor, while leupeptin is a reversible and competitive intralysosomal proteolysis inhibitor that specifically blocks serine and some cysteine proteases. As revealed by Western blotting, treatment with MG-132 was able to significantly restore CD48 expression (Figure 6D). We also observed however, that the presence of leupeptin partially abrogated CD48 degradation. Consistent with these findings, immunofluorescence microscopy assays evidenced enhanced CD48 signals in the wt MCMV-infected macrophages exposed to the two different proteolysis inhibitors (Figure 6E, panels j and n) as compared to that of the untreated-infected cells (Figure 6E, panel f). Moreover, co-localization of m154 and CD48 could be clearly visualized in both MG-132 and leupeptin treated-infected macrophages (see panels l and p in Figure 6E). Altogether, the data indicate that MCMV targets CD48 for degradation, likely using both the proteasome- and the lysosome-mediated mechanisms.

### Absence of m154 augments the susceptibility of MCMV-infected macrophages to NK cell-mediated degranulation

**Figure 7:**
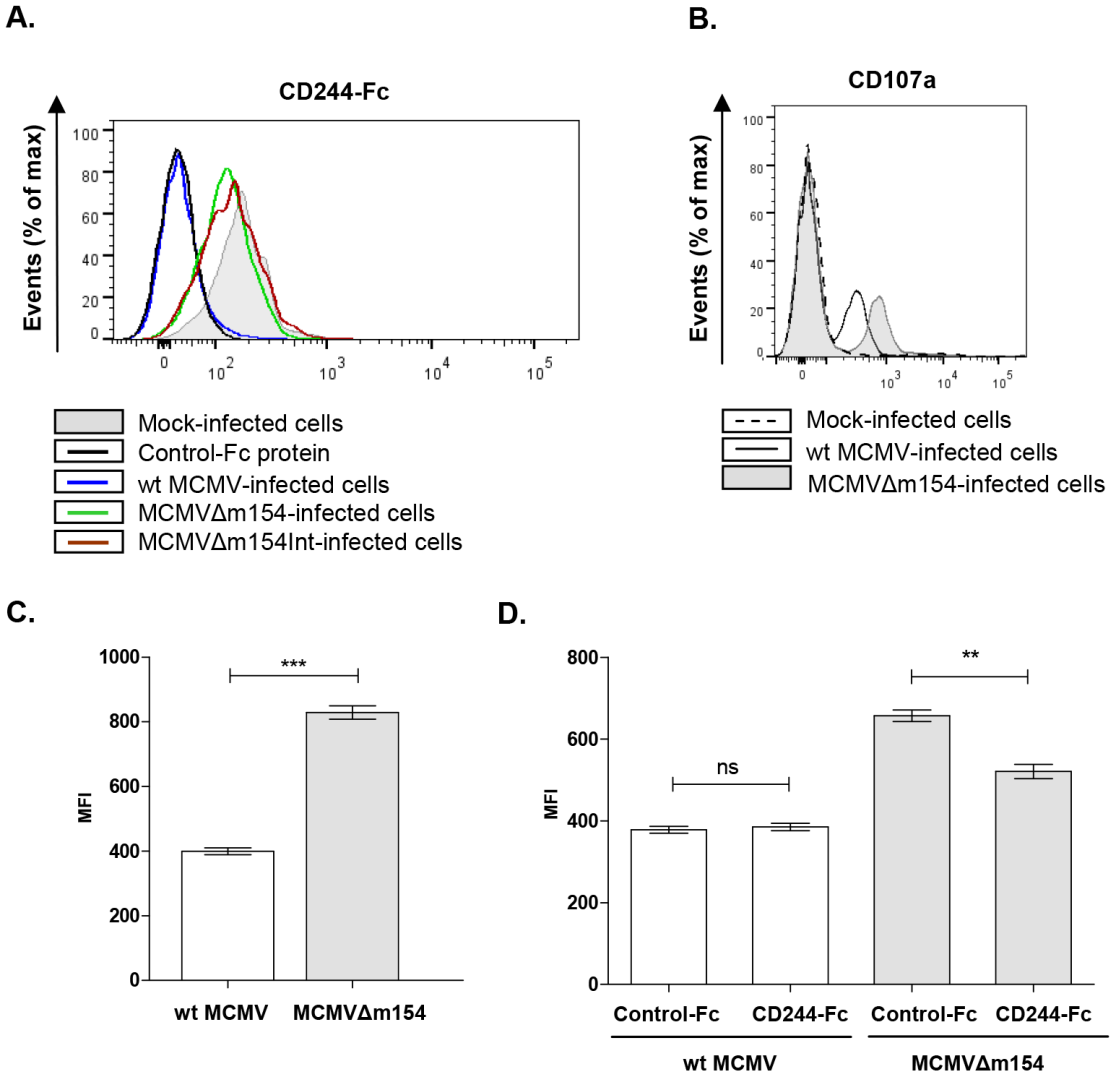
m154-mediated downregulation of CD48 in the infected cell diminishes CD244 recognition and provokes impaired NK degranulation. (A) Peritoneal macrophages, mock-infected (shaded histogram), infected with wt MCMV (black line histogram), MCMVΔm154 (dotted line histogram) or with MCMVΔm154Int (gray dashed line histogram) at an moi of 10 were labelled with 1 µg of murine biotinylated CD244-Fc protein followed by streptavidin-PE. An unrelated biotinylated Fc fusion protein (black dashed line histogram) was used as a control. One representative experiment out of three is shown. (B) Representative flow cytometry histograms of CD107a expression on DX5^+^ NK cells incubated with mock-infected (dotted histogram), wt MCMV (black line histogram) or MCMVΔm154 (shaded histogram) infected cells (moi 10) at a E/T ratio of 1∶1 (C) Graph of a representative experiment out of three showing the mean (± SEM) of MFI of CD107a^+^ NK cells incubated with wt MCMV (empty bar) or MCMVΔm154 (shaded bar) infected cells as indicated in B. ***p<0.001. (D) Peritoneal macrophages were mock-infected or infected with MCMVΔm154 at an moi of 10. Infected cultures were incubated with 10 µg/ml of murine CD244-Fc or of an unrelated Fc fusion protein used as a control. Cultures were washed and exposed to the NK cells at a E/T ratio of 0.5∶1. Graph of a representative experiment out of three showing the mean (± SEM) of the normalized percentage of CD107a^+^ NK cells for each sample referred to the number of CD107a^+^ NK cells in MCMVΔm154-infected macrophages in the absence of fusion protein. **p<0.01.

As indicated, the natural ligand for CD48 is CD244, a molecule that is expressed on all NK cells, and to a lesser extent, on other cytotoxic leukocytes. We first sought to explore whether infection of macrophages with MCMV resulted in a reduced recognition by CD244 due to the loss of CD48 on the cell surface. For this purpose, we generated a soluble murine CD244-Fc fusion protein containing the ectodomain of CD244 fused to the Fc portion of the human IgG. As shown in Figure 7A, binding of CD244-Fc fusion protein to macrophages was significantly decreased upon wt MCMV infection. On the other hand, the fusion protein interacted with MCMVΔm154- and MCMVΔm154Int-infected cells in a similar manner to non-infected cells (Figure 7A).

The interaction of CD48 with CD244 increases NK cell activation, triggering cytotoxicity. Thus, by suppressing CD48-surface expression, m154 could help MCMV elude NK cell-mediated immune responses. To ascertain whether this was the case, we compared the degranulation capacity of NK cells after exposure to macrophages infected with wt MCMV or MCMVΔm154. For this purpose, we used a flow cytometric-based assay to measure NK cell-surface expression of LAMP-1 (CD107a). NK cells purified from mouse spleens were incubated with mock-, wt MCMV- or MCMVΔm154-infected cells at a macrophage/NK ratio of 1∶1. As expected, the percentage of CD107a^+^ NK cells specifically augmented in response to the viral infection as compared to non-infected cultures. No substantial differences could be detected, however, in any of the experiments performed, when we compared the percentage of CD107a^+^ NK cells incubated with wt MCMV and those incubated with mutant MCMV-infected macrophages (e.g. mock: 5.5%±1.7; wt MCMV: 31.6%±2.9; MCMVΔm154: 26.3%±1.3). In contrast, we observed markedly increased CD107a externalization in the NK cell population responding to the *m154* defective MCMV, as indicated by a 2-fold increase in the CD107a mean fluorescence intensity (MFI) on the CD107a^+^ NK cells exposed to MCMVΔm154-infected cultures as compared to wt MCMV (Figure 7B and 7C). Thus, the mean number of granules discharged by individual degranulating NK cells during stimulation by MCMV-infected macrophages was lower when m154 was being expressed. To evaluate whether the effects on NK-cell responses observed were caused by m154 acting on the CD48/CD244 axis, we performed degranulation assays on co-cultures of NK cells and MCMVΔm154-infected cells pre-incubated with the CD244-Fc fusion protein. As shown in Figure 7D, the CD244-Fc fusion protein partially blocked CD107a surface expression on NK cells exposed to the MCMVΔm154-infected cells, while an irrelevant control Fc fusion protein did not have a significant impact. Together, the results indicate that m154 contributes to confer protection to MCMV-infected macrophages against NK cell attack, and that these effects are mediated, at least in part, through m154 downregulation of CD48.

### m154 enhances *in vivo* MCMV growth by protecting against NK cell-mediated control

**Figure 8:**
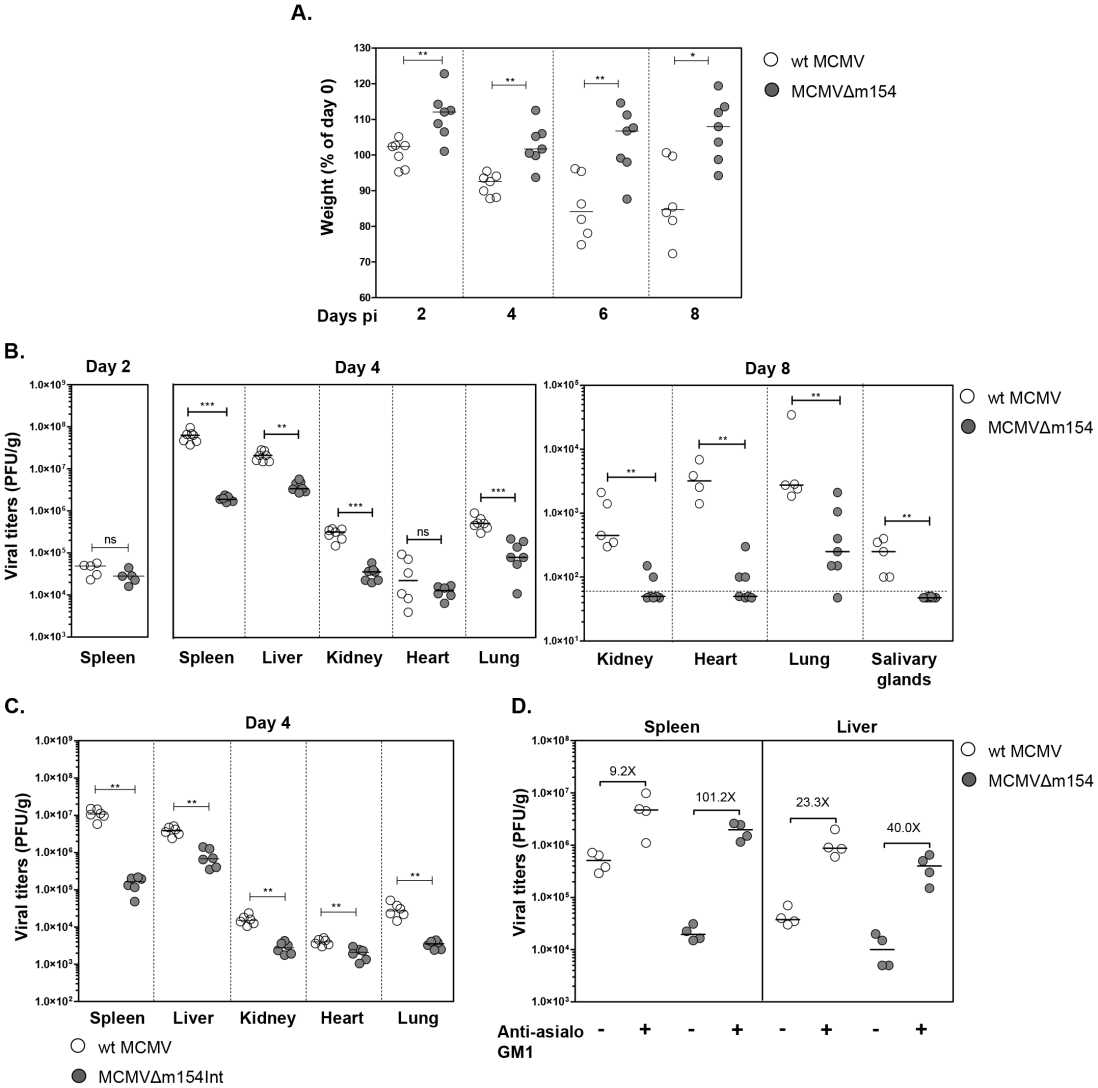
*In vivo* attenuation of MCMVΔm154 in an NK cell-dependent manner. (A) Groups of 7-weeks-old BALB/c.ByJ female mice were i.p. inoculated with 2×10^6^ PFU of wt MCMV (open circles) or MCMVΔm154 (filled circles). Changes in the percentage of body weight relative to day 0, measured at days 2, 4, 6 and 8 post infection (pi), are shown. (B) Mice infected as indicated in A were sacrificed at days 2, 4 and 8 after infection and viral titers from selective organs determined. The horizontal dashed line marks the limit of detection. (C) Groups of 7-weeks-old BALB/c.ByJ female mice were i.p. inoculated with 2×10^6^ PFU of wt MCMV (open circles) or MCMVΔm154Int (filled circles). Mice were sacrificed at day 4 after infection and viral titers from the indicated organs determined. (D) Depletion of NK cells restores MCMVΔm154 *in vivo* phenotype. Groups of 5-week-old BALB/c.ByJ female mice were injected i.p. with anti-asialo GM1 antibody or left untreated. Animals were i.p. inoculated with 5×10^5^ PFU of wt MCMV (open circles) or MCMVΔm154 (filled circles), sacrificed at day 4 after infection and viral titers in spleen and liver determined. Values corresponding to differences in median values between animals infected with the same virus, untreated or treated with anti-asialo GM1 antibody, are shown. *p<0.05, **p<0.01, ***p<0.001, ns: non significant. Each symbol represents an individual mouse. Horizontal bars indicate the median values.

We reasoned that reduction of CD48 surface expression on antigen-presenting cells may contribute to the host's impaired ability to control viral growth. We therefore sought to explore the impact that m154 plays in the context of an acute viral infection by inoculating BALB/c mice with MCMVΔm154 or wt MCMV. By day 2 after infection, we could observe that mice intraperitoneally (i.p.) inoculated with 2×10^6^ plaque forming units (PFU) of MCMVΔm154 had gained a larger percentage of body weight than mice infected with the same dose of wt MCMV (Figure 8A). Moreover, while wt MCMV-infected animals lost a substantial percentage of body weight by days 4, 6 and 8 after infection, animals infected with MCMVΔm154 did not experienced any weight loss during the course of the assay. Thus, at day 8 after infection the average body weight of mice infected with wt MCMV was 14.4 g±4.0, whereas mice infected with MCMVΔm154 had an average body weight of 18.3 g±4.3 (data not shown). In agreement with the loss of body weight, wt MCMV-infected mice also developed more exacerbated clinical signs of disease, such as ruffled hair, hunched posture and lethargy (data not shown). When we analyzed the frequency of infected peritoneal macrophages, we did not find significant differences between wt MCMV- and MCMVΔm154-infected mice (wt MCMV: 3.0%±0.6; MCMVΔm154: 2.9%±0.2). Neither, the nature of the cellular influx to the peritoneal cavity, as determined by the levels of neutrophils (CD11b^+^ Gr-1^+^), macrophages (CD11b^+^ Gr-1^−^), T lymphocytes (CD3^+^) or B lymphocytes (IgM^+^) appeared to be distinct amongst the two groups of infected animals (Figure S1). We subsequently determined the replication levels of the viral deletion mutant in several target organs of the animals at different days post-infection. As depicted in Figure 8B, while at day 2 post-infection, comparable viral titers were observed in the spleens of wt MCMV- and MCMVΔm154-infected mice, at day 4 after infection, viral titers in MCMVΔm154-infected animals were around 32-, 6-, 9-, and 4- fold lower in spleen, liver, kidney, and lung, respectively, than those found in the same organs of wt MCMV-infected mice. Likewise, at day 8 post-infection, viral loads of MCMVΔm154 were considerably lower in the organs analyzed (kidney, heart, lung, and salivary glands) compared to those of wt MCMV. Indeed, at this time point, viral titers were below the assay's detection limit in a number of the MCMVΔm154-infected animals (Figure 8B). Comparable results were obtained when mice infected with MCMVΔm154Int were analyzed at day 4 post-infection (Figure 8C). Thus, we can conclude that MCMVs lacking the *m154* gene are attenuated in all major organs targeted during MCMV infection.

These results, showing the *in vivo* effects of m154 as early as 4 days post-infection, together with the *in vitro* data pointing to a contribution of m154 impairing NK degranulation against the infected macrophages, were highly indicative of an MCMV evasion mechanism involving NK cell immune surveillance. Therefore, we decided to examine whether the reduced attenuation of MCMVΔm154 was a consequence of its enhanced susceptibility to NK cells during the *in vivo* infection. Mice were specifically depleted of NK cells by treatment with rabbit antiserum to asialo GM1. Four days after infection with 8×10^5^ PFU of wt MCMV or MCMVΔm154, mice were sacrificed and assayed for viral loads in spleen and liver, the two predominant organs in which NK cells have been reported to intervene in the control of MCMV. As expected, all mice treated with anti-asialo GM1 antibody had significantly higher viral titers in their spleens and livers as compared to the corresponding untreated control mice (Figure 8C). However, the extent to which these viral titers were elevated following NK ablation was considerably superior in the MCMVΔm154-infected animals (in particular in the spleen, 101-fold) than in animals infected with wt MCMV (9-fold in spleen). Thus, this substantial restoration of MCMVΔm154 replication demonstrates that m154 promotes MCMV growth *in vivo* by subverting NK cell responses.

## Discussion

For an effective immune response against many viral infections, antigen-presenting cells such as dendritic cells and macrophages must expose a concerted repertoire of receptors that alert T and NK cells for their efficient activation. In this context, distortion of the surface receptor content is a maneuver widely adopted by numerous viruses to elude the immune system and secure an optimal milieu for their replication and dissemination. In this study we show that several cell-surface molecules of the SLAM family, which operate as co-signalling molecules triggering distinct signal-transduction networks in T, NK and antigen-presenting cells, are targeted by MCMV. Notably, CD48, CD84, CD229 and Ly108 get differentially restricted from the cell surface within the window of time it takes for the virus to complete its life cycle and produce productive progeny. Hence, the fact that CMV might have an active interest in interrupting SLAM interactions through the downregulation of the specific receptors/ligands in the infected cell indicates that, at least for the four SLAM members analyzed in our study, engagement of the corresponding receptors/counter receptors should exert prevailing activating signals in key immune cells during infection.

In this study, we decided to further explore in more detail the loss of CD48-surface expression after MCMV infection. CD48, a GPI-anchored molecule with broad expression in hematopoietic cells, is a SLAM receptor not involved in the homophilic interactions distinctive of this family, its natural ligand being CD244 [17]. Accordingly, reduction of CD48 from the surface of MCMV-infected macrophages leads to a drastic decrease in CD244 binding compared to that observed in mock-infected cells. By screening a battery of MCMV deletion mutants, we identified m154 as the viral downregulator of CD48. Thus, deletion of both the complete *m154* sequence or of an internal part of this viral gene from the MCMV genome is sufficient for restoring the surface levels of CD48 back to those found in non-infected cells. Moreover, m154 ectopically expressed within the MCMV genome leads to a significant decrease of CD48 on the surface of the infected macrophage. The *m154* ORF encodes a type I transmembrane protein containing a remarkable mucin-type extracellular region. By generating a specific mAb (m154.4.113) against the cytoplasmic tail of this protein, we found that m154 expression is initiated in the early phase of infection and continues throughout the infection cycle, a time frame that is concomitant with the progressive downregulation of CD48 in the infected macrophage. In addition, we show that this viral protein preferentially localizes on the surface of the infected cell.

m154 belongs to the m145 family of glycoproteins [35], despite not presenting the MHC class I protein fold characteristic of some family members. Notably, several of the ten members (m17, m145 to m158) that comprise this family have been reported to perform immunoevasive activities [37]. In particular, the m145, m152, and m155 proteins each downregulate one or more ligands of the activating NK cell receptor NKG2D (H60, RAE1, or MULT-1; [38]–[42]). Additionally, m152 causes intracellular retention of MHC class I molecules [43], while m155 reduces cell-surface expression of the costimulatory molecule CD40 [44]. Finally, the m157 protein interacts with Ly49 NK cell receptors and engages both NK activating (Ly49H) and inhibitory receptors (Ly49I) [36], [45], [46]. Thus, m154 can be now added to the group of molecules within the m145 family that operates as an immunoevasin. While it is important to point out that m154 is able to selectively downregulate CD48, since surface molecules like CD86, Ly108, or CD84, which are used as specificity controls, are not affected by m154, we can not exclude, however, that this viral protein also has a multifunctional nature and targets additional immune receptors.

In terms of delineating the mechanism that leads to the loss of surface CD48, we determined that it does not occur at transcriptional level. Instead, our data show that m154 appears to be majorly causing CD48 degradation. The viral protein leads to a major reduction in the total cellular amount of CD48. Through Western blot analysis and immunofluorescence microcopy, we found that treatment of infected cells with either the serine and cysteine protease inhibitor leupeptin or the proteasome inhibitor MG-132 stabilizes CD48 expression in a certain degree, suggesting that both the lysosomal and proteasomal degradation pathways play a role in the downregulation of CD48. In addition, using these proteolysis inhibitors, co-localization of m154 and CD48 could be appreciated in the wt MCMV-infected macrophages. Interestingly, the m154 cytoplasmic tail displays a motif that has been implicated in lysosomal targeting, and two overlapping recognition sites for the adaptor protein AP-2, which is involved in clathrin-dependent endocytosis. While the detailed mechanism by which m154 operates remains to be elucidated, it does not seem to involve the overall trafficking of GPI-linked receptors, as cell-surface expression of other GPI-anchored membrane molecules, such as CD55, are not affected by this viral protein (data not shown).

m154 does not have a counterpart in any other of the CMV species whose genome has been sequenced so far. However, akin to MCMV, we have previously reported that CD48 is also downregulated in HCMV-infected macrophages [47]. Therefore, each CMV might have evolved its own CD48-specific inhibitor, as yet to be identified for HCMV, emphasizing the importance of targeting this molecule to evade NK cell recognition during infection. In addition, and similarly to MCMV, we found that other SLAM receptors are markedly reduced from the cell surface of macrophages upon HCMV infection (Angulo, unpublished observations). Whether the overall downmodulation of SLAM receptors in the infected cell is an inherent and unique property of CMVs, reflecting selection pressures faced in their specific niches, or whether this might be used by other viruses as an immune evasion mechanism, remains to be explored. Notably, CD48 and NTB-A have been also reported to be negatively regulated by HIV, leading to impaired NK cell recognition and lysis of the infected CD4^+^ T cells, and being the viral accessory protein Vpu identified as the NTB-A downregulator [27], [48].

Increasing evidence indicates that CD244 contributes to the regulation of both NK cell antiviral activity and virus-specific CD8^+^ T cell functionality in humans and mice [49]–[51]. Engagement of CD244 by CD48 in the NK cell results in the recruitment and clustering of the receptor into lipid rafts, the phosphorylation of the ITSMs within its intracellular tail, and the subsequent association with the adapter molecule SAP [18], [52]–[54]. This triggers a signalling cascade, leading to the formation of the NK cell synapse, which is characterized by the polarized release of cytolytic granules containing perforin and granzymes. The NK cell synapse is most likely critical for activated NK cells to interact in a productive manner with MHC class I-negative target cells and induce potent cell cytotoxicity [55]. On the other hand, CD244 can inhibit NK cell activation in the absence of functional SAP, such as occurs in cells from patients with X-linked lymphoproliferative syndrome [56]. Taken together, these observations indicate that CD244 and SAP modulate the activity of normal NK cells. Here, we specifically show that disruption of the *m154* gene in MCMV leads to an enhanced antiviral NK cell response *in vitro*. In particular, this viral protein limits NK degranulation capacity against MCMV-infected cultured macrophages. Moreover, we present that the NK cell response to cells infected with the MCMV lacking the *m154* gene can be partially inhibited by preincubation of the infected macrophages with the CD244-Fc fusion protein. Hence, we infer from these results that by downregulating CD48, m154 may help protect MCMV-infected cells from NK killing. We cannot discard the possibility, however, that m154 might be also be capable of exerting other functions that contribute to these effects.

As expected, the *m154* gene is not required for replication *in vitro* and an MCMV lacking *m154* has not an altered growth phenotype in cultured MEFs or macrophages. It is of note that parental and mutant viruses used throughout the study do all derive from MCMV-BAC pSM3fr, containing an mck-2 frameshift mutation associated with reduced ability to infect macrophages and a diminished capacity to attract leukocytes [57], [58]. However, the fact that all of these recombinant MCMVs have the same pSM3fr background makes them comparable at the level of the MCK-2 phenotype. In contrast to the *in vitro* observations, the severity of the infection of viruses that do not express m154 was significantly impaired *in vivo*, where they exhibited a substantial restricted growth in all organs analyzed. By day 4 post-infection, the differences in splenic and liver growth between wt MCMV and MCMVΔm154 were around 30- and 10-fold, respectively, consistent with m154 counteracting NK cell responses, which are crucial to the early control of MCMV replication. Accordingly, we show that mice depleted of NK cells with an antibody to asialo GM1, a glycosphingolipid present at high concentrations in this cell population, selectively improved the *in vivo* replication of the deletion mutant, confirming that the mechanism by which m154 exerts its protective role is NK cell dependent.

Because CD244 expression is not restricted to NK cells, the impact of m154 might have implications that extend beyond the regulation of NK cell function. This receptor is also present at lower levels on other cytotoxic cells, such as CD8^+^ T cells, γδ T cells, basophils, and eosinophils. In particular, upon interaction with CD48, CD244 helps initiate signalling and cellular cytotoxicity in CD8^+^ T cells [49]. Hence, one could speculate that other non-NK cell-related mechanisms might be also contributing to the net protective effects of m154 *in vivo*. However, the fact that NK cell depletion results in the near complete rescue of the *m154*-deleted MCMV growth phenotype *in vivo* indicates that, at least under the conditions of early acute infection analyzed here, the potential of this viral protein to influence processes mediated by other immune cell subsets might be relatively minor. It remains to be determined whether additional effects of m154 could be of relevance later during the infection or in other scenarios the virus might encounter.

In summary, our study presents the SLAM family of immunoreceptors as a novel target of manipulation by CMV, adding to the diversity of molecular strategies incorporated by this pathogen to escape immune detection. We have identified for the first time a herpesviral gene implicated in the downregulation of the SLAM member CD48, and documented its protective role *in vivo* by counteracting NK cell responses. The knowledge gained from the findings reported in this manuscript will contribute to a better understanding of the complex host-CMV interactions and provide additional insights into the functioning of the SLAM receptors in viral immunity. Finally, the identification of novel players that increase the CMV burden early on during infection could prove helpful for the future development of antiviral reagents.

## Materials and Methods

### Ethics statement

All procedures involving animals and their care were approved (protocol number CEEA 308/12) by the Ethics Committee of the University of Barcelona (Spain) and were conducted in compliance with institutional guidelines as well as with national (Generalitat de Catalunya decree 214/1997, DOGC 2450) and international (Guide for the Care and Use of Laboratory Animals, National Institutes of Health, 85-23, 1985) laws and policies.

### Cells

The cell lines NS-1 (mouse myeloma) and 300.19 (mouse pre-B) were obtained from the American Type Culture Collection. Cells were grown in RPMI 1640 medium (GIBCO-BRL, Paisley, UK) supplemented with 10% fetal bovine serum (Sigma Aldrich, St. Louis, MO), 100 U/ml penicillin, 100 U/ml streptomycin, 1 mM sodium pyruvate, and 2 mM L-glutamine (GIBCO-BRL). 300.19 cells were maintained in media supplemented with 0.05 mM 2-mercaptoethanol (GIBCO-BRL). Primary mouse embryonic fibroblasts (MEFs) were cultured in Dulbecco's modified Eagle's medium (DMEM; GIBCO-BRL) supplemented as indicated above. Primary macrophages were elicited from peritoneal exudate cells (PECs) following i.p. injection of 1 ml of 3% thioglycollate (Sigma Aldrich) into BALB/c mice. PECs were removed by peritoneal lavage. Cells were plated out at 2×10^5^ cells/ml in supplemented RPMI 1640 medium, and incubated for 2 h at 37°C, 5% CO_2_, after which nonadherent cells were washed away with phosphate buffered saline (PBS). Macrophage preparations were confirmed by flow cytometry using the markers F4/80 and CD11b (about 95% were F4/80^+^CD11b^+^). NK cells were obtained from mouse spleen using the mouse NK cell isolation kit II (Miltenyi Biotec, Bergisch Gladbach, Germany) on an AutoMACS (Miltenyi Biotec).

### Viruses

The BAC-derived MCMV, MW97.01, based on the MCMV Smith strain (ATCC VR-1399) and referred to here as wt MCMV [59], and the MCMV-GFP recombinant virus, a derivative of MW97.01 carrying the GFP gene [32] were used as parental viruses throughout the study. Recombinant strains MCMV-GFPΔ6 lacking genes from *m144* to *m158* (referred to here as MCMV-GFPΔm144-m158), MCMV-GFPΔ6S1 lacking genes from *m144* to *m148* (referred to here as MCMV-GFPΔm144-m148), MCMV-GFPΔ6S2 lacking genes from *m149* to *m153* (referred to here as MCMV-GFPΔm149-m153), MCMV-GFPΔ6S3 lacking genes from *m154* to *m157* (referred to here as MCMV-GFPΔm154-m157), MCMV-GFPΔm153-m154 lacking genes *m153* and *m154* (referred to here as MCMV-GFPΔm153-m154), and MCMV-Dm155Dm157FRT lacking genes from *m155* to *m157* (referred to here as MCMV-GFPΔm155-m157) have been described previously [33], [40]. For the generation of recombinant MCMV lacking *m154* ORF (referred to here as MCMVΔm154) a kanamycin resistance (KanR) cassette was amplified from the plasmid pGP704 with primers Dm154Fw (5′- CCC GCC AAT CAC ATT CAC GAG GGG GTG CTC CGA GAT ACG GTC TCG ACC ACA GGA CGA CGA CGA CAA GTA -3′) and Dm154Rv (5′- CAC ATA AGA CTC GTC ATA ACC TTC CCC GAG TGC CAC CTC CCC ACC CTT ATC GTC TCA GGA ACA CTT AAC -3′) (underlined letters are homologous to the MCMV genome). For the generation of recombinant MCMV lacking only ORF *m154* and without affecting ORFs *m154.3* and *m154.4* described in Tang *et al.* [34], referred to here as MCMVΔm154Int, a KanR cassette was amplified from the pGP704 with following primers: Dm154bFw (5′- CCG CTG CGG ACG CGA TCT CTT CGG CAA CCC CTA GTG CAG GTG CCG TTA GGA CGA CGA CGA CAA GTA A -3′) and Dm154Rv. PCR fragments were inserted into the *m154* ORF of the MCMV BAC MW97.01 by red-α, -β, -γ-mediated recombineering [60]. Subsequently, the KanR cassette was excised by FLP recombinase. For MCMVm154Ectop, the *m154* ORF plus sequences containing the putative promoter and the polyadenylation signal were PCR amplified using primers m154-ek.for (5′- CGC GTT AAC CCC GTA TAA ACA CCG CAC CAG A -3′) and m154-ek.rev (5′- CGC AGA TCT ATG TCC TGA CAG ATT ATC GTG GT -3′) with MW97.01 DNA as a template. The PCR product was cloned into pOri6K-F5 [61] and then the *m154* sequences together with the adjacent KanR cassette (flanked by mutant [F5] FRT sites) was amplified using primers m154-ins.for (5′- AAC CAC GGG TTC TTT CTC TTG ACC AGA GAC CTG GTG ACC GTC AGG AAG AAG ATT CAG TGA CAG GAA CAC TTA ACG GCT GA -3′) and m154-ins.rev (5′- GTC CGA TGA ATA AAA CCT CTT TAT TTA TTG ATT AAA AAC CAT GAC ATA CCT CGT GTC CTC CCC GTA TAA ACA CCG CAC CA -3′). The *m154* sequences and the KanR cassette were inserted downstream of the *ie2* (*m128*) ORF of the MCMVΔm154 BAC by red-α, -α, -γ-mediated recombineering followed by excision of KanR as described above. The integrity of the MCMVΔm154, and MCMVΔm154Int, and MCMVm154Ectop genomes was verified by restriction analysis and sequencing. Viral stocks were prepared by infecting MEFs at low moi. Cell supernatants were recovered when maximum cytopathic effect was reached, and cleared of cellular debris by centrifugation at 1.750× g for 10 min. Viral titers were determined by standard plaque assays on MEFs. Peritoneal macrophages were infected with parental MCMV, MCMV-GFP or derived deletion mutants at an moi ranging from 0.2 to 10. Adsorption was for 2 h at 37°C, 5% CO_2_, including a centrifugal enhancement of infectivity step [62]. Cells were then washed in PBS before fresh medium was added. The percentage of macrophages infected by recombinant viruses not expressing GFP was estimated by indirect immunofluorescence 24 h after infection using the anti-MCMV IE1 mAb Croma 101 followed by goat anti-mouse IgG Alexa Fluor-488. UV-inactivation of virus was performed using a UV crosslinker (HL 2000 hybrilinker, UVP [254 nm UV], Upland, CA) for 3 min at 360 mJ/cm^2^.

### Antibodies

The anti-m154 mAb (clone m154.4.113, IgG_2a_) was generated by fusing an NS-1 myeloma cell line with spleen cells from a BALB/c mouse immunized three times with the synthetic peptide corresponding to the intracellular tail of m154 (HRWEDDKGGEVALGEGYDESYV) conjugated to KLH (Proteogenix, Oberhusbergen, France). The hybridoma was subcloned at least three times. The following mAbs were obtained from Biolegend (San Diego, CA): anti-mouse CD48-PE, anti-mouse CD48-Alexa Fluor 488, CD84-PE, Ly108-PE, H2-PE, CD150-PE, CD86-PE, F4/80-pacific blue, CD49b-PE/Cy7, CD107a-Alexa Fluor 488, CD3-Alexa Fluor 647, and streptavidin-PE. Biotin anti-mouse 2B4, recognizing mouse CD244, anti-mouse CD229-APC, CD11b-PE, and Gr-1-APC were purchased from Becton Dickinson Bioscience (San Diego, CA), anti-mouse CD48 (HM48-1) from Santa Cruz Biotechnology (Santa Cruz, CA), and IgM-FITC from Southern Biotech (Birmingham, AL). Anti-mouse IgG Alexa fluor 555 and anti-mouse IgG-Alexa Fluor 488 were purchased from Invitrogen (Carlsbad, CA). Anti-rabbit IgG and anti-Armenian hamster IgG (H+L) labelled with horseradish peroxidase (HRP) were obtained from Jackson Immuno Research Laboratories (West Grove, PA), and anti-mouse IgG (H+L) labelled with HRP from Promega (Heidelberg, Germany). Biotin-conjugated anti-HA and anti-mouse β-actin were purchased from Sigma Aldrich. Isotype control IgGs directly conjugated with the corresponding fluorochromes were obtained from Immunotools (Friesoythe, Germany). The MCMV IE1 specific mAb Croma 101 has been previously described [63].

### Plasmid constructions and transfections

A murine CD244-Fc fusion protein containing the CD33 leader peptide and the Fc region of human IgG_1_ was obtained by inserting sequences corresponding to the CD244 ectodomain into the mammalian expression vector signal pIg-Tail (R&D Systems, Wiesbaden, Germany) as previously described [64]. The construct corresponding to the Ig fusion protein was subcloned into the expression vector pCI-neo. An NS-1 stable transfectant secreting the CD244-Fc fusion protein was obtained by electroporation and selection using 1.2 mg/ml geneticin (G418) (GIBCO-BRL). Eight million cells were electroporated (280V, 950 µF) with 8 µg of linearized DNA using the Gene Pulser II Apparatus (Bio-Rad, Hercules, CA). The transfected cells were plated in flat-bottom, 96-well tissue culture plates (Costar, Corning, NY) by limiting dilution and the clone producing the highest amounts of fusion protein was cultured in INTEGRA CL 1000 flasks (Integra Biosciences AG, Chur, Switzerland). The supernatant containing the fusion protein was purified using the Affi-Gel Protein-A MAPS II kit (Bio-Rad). An m154 N-terminal HA fusion protein (HA-m154) was constructed using a PCR product of m154 obtained with primers m154BglII (5′- CCA AGA TCT TTG GGT CGT TTA GAG CTT -3′) containing a *Bgl*II restriction site, and m154PstI (5′- CTC CTG CAG TCA CAC ATA AGA CTC GTC -3′) containing a *Pst*I restriction site, and DNA extracted from MCMV virions as a template. The PCR product was inserted into the pGEMT vector and a *Bgl*II-*Pst*I fragment corresponding to the *m154* gene without the signal peptide was then excised and inserted in frame with the HA at the N-terminal end of the m154 protein into the mammalian expression vector pDisplay (Invitrogen) treated with *Bgl*II and *Pst*I. 300.19 stable transfectants were obtained using the same protocol as indicated for NS-1 cells, except that plasmids expressing HA-m154 or the corresponding empty pDisplay vector were transfected using the Amaxa Cell Line Nucleofector Kit V (Amaxa AG, Koeln, Germany) according to the manufacturer's protocol, and selection performed with 0.8 mg/ml of G418.

### Flow cytometry analysis

Flow cytometry was performed using standard procedures. Fc fusion protein stainings were performed using 2 µg of biotinylated Ig fusion protein followed by incubation with streptavidin-PE. Samples were analyzed using a FACSCanto II (BD Biosciences) flow cytometer and processed with the accompanying FlowJo software (Tree star Inc, Ashland, OR). Ten thousand cells were counted for each sample. Cell viability was measured using the LIVE/DEAD Fixable Violet Dead Cell Stain Kit (Invitrogen) according to the manufacturer's instructions. The FACSCalibur (BD Biosciences) was used for analysis of the cellular influx to the peritoneal cavity.

### RT-PCR

Total RNA was isolated from peritoneal macrophages either uninfected or MCMV-infected for 72 h by the TRIzol method (Invitrogen). RT-PCR was then carried out using the SuperScript III First-strand Synthesis System for RT-PCR (Invitrogen) according to the manufacturer's protocol. Briefly, RNA samples were treated with RNase-free DNase I (Promega) for 30 min at 37°C, and the DNase was inactivated at 65°C for 10 min. The RNA was reverse transcribed using oligo(dT) primers at 50°C for 50 min, and reactions were terminated by heating at 85°C for 5 min. The reverse-transcribed products were treated with RNase H for 20 min at 37°C and amplified using specific primers. Primers m154For (5′- CTT GGA TCC ATG CGG GCG ATG TTA CGG -3′) and m154Rev (5′- CTC GGA TCC CAC ATA AGA CTC GTC ATA -3′) were used to amplify a 1116-bp fragment within the MCMV *m154* gene, primers mCD48For (5′- ATG TGC TTC ATA AAA CAG GG -3′) and mCD48Rev (5′- TTG TCA GGT TAA CAG GAT CCT GTG -3′) were used to amplify a 726-bp fragment within the murine *CD48* gene, and primers β-actinFor (5′- TAT CCT GAC CCT GAA GTA CC -3′) and β-actinRev (5′- TCA TCT TTT CAC GGT TGG CC -3′) were used to amplify a 170-bp fragment within the murine β-actin gene. PCRs were performed under the following conditions: 1 cycle at 94°C for 5 min; 30 cycles of 1 min at 94°C, 1 min at 58°C, and 1 min at 72°C; and 1 cycle at 72°C for 10 min. Control reactions carried out in the absence of RT were used to assess the specific detection of RNA. Amplified products were separated on a 1% agarose gel and visualized by ethidium bromide staining.

### Western blot analysis

For Western blot analysis, peritoneal murine macrophages, either mock-infected or infected with MCMV at an moi of 10 were used. For selective expression of viral immediate-early proteins, cells were incubated from 30 min prior to infection to 4 h post-infection in the presence of CHX (100 µg/ml; Sigma Aldrich), followed by incubation in the presence of actinomycin D (10 µg/ml; Sigma Aldrich) for another 12 h. Selective expression of early genes was carried out by treatment of the cells with PPA (250 µg/ml; Sigma Aldrich) for 72 h. For proteolysis inhibition experiments, cells were treated with 75 µM MG-132 (Sigma Aldrich) or 250 µM leupeptin (Sigma Aldrich) for 6 h and 24 h, respectively, before harvesting. Under the conditions used, MG-132 and leupeptin did not generally affect cell viability as assessed by trypan blue cell staining. At the indicated times after infection for each specific case, samples were lysed in protein sample buffer and boiled for 5 min. Cell lysates were subjected to SDS-PAGE in 10% acrylamide gels and subsequently transferred to nitrocellulose membranes (Protran, Whatman Schleicher & Schuell, Germany). Equal quantities of total protein were analyzed per lane. Membranes were probed using the mAb anti-m154 (clone m154.4.113), mAb anti-IE1 Croma 101, and an anti-mouse IgG (H+L) HRP as a secondary antibody, and mAb anti-mouse CD48 (HM48-1) followed by a HRP-conjugated goat anti-Armenian hamster IgG (H+L) antibody. β-actin was detected using the mAb anti-β-actin and an HRP-conjugated goat anti-rabbit IgG as a secondary antibody. Blots were developed using a SuperSignal West Pico Chemiluminescent Substrate (Pierce, Rockford, IL) according to the manufacturer's protocol.

### Immunofluorescence microscopy

Peritoneal murine macrophages, mock-infected or MCMV-infected at different mois, were cultured on glass coverslips in 24-well tissue culture plates. When indicated, cultures were exposed to proteolysis inhibitors as indicated for the Western blot analysis. At specific time points after infection, the cells were washed in PBS and fixed and permeabilized using ice-cold methanol and 0.3% Triton X-100 (for IE1 detection), or fixed in ice-cold acetone (for m154 and CD48 detection), and subsequently blocked with 1% bovine serum albumin (Sigma Aldrich; for IE1 detection) or with 20% rabbit serum (Linus) and 6% fetal bovine serum in PBS (for m154 and CD48 detection). The cells were stained with anti-m154 mAb (clone m154.4.113), or with MCMV IE1 mAb Croma 101, using as secondary antibodies a goat anti-mouse IgG (H+L) Alexa Fluor 555 or Alexa Fluor 488, or directly with anti-mouse CD48-Alexa Fluor 488. Nuclei were counterstained with DAPI reagent (Invitrogen). The samples were mounted in ProLong Gold antifade reagent (Invitrogen). Fluorescence images were obtained using a Nikon Eclipse E600 microscope (Nikon, Tokyo, Japan) or an inverted Leica DMI6000B microscope and the LAS AF software from Leica Microsystems (Wetzlar, Germany).

### Immunoprecipitation

Peritoneal macrophages were surface-labeled with biotin (Sigma Aldrich) and lysed in protein sample buffer. Cell lysates were precleared 3 times for 30 min using protein G Sepharose (GE Healthcare) and incubated overnight with anti-m154 mAb and protein G Sepharose. Immunoprecipitates were washed, eluted, subjected to SDS-PAGE in 10% acrylamide gels and transferred to nitrocellulose membranes. Membranes were probed with streptavidin-POD conjugate (Roche Diagnostics GmbH, Mannheim, Germany) and blots developed as for the Western blot analysis.

### Determination of MCMV replication kinetics

Multi-step growth *in vitro* was analyzed by infecting MEFs or peritoneal macrophages in 24-well plates with wt MCMV or MCMVΔm154 at an moi of 0.025 and 0.2, respectively. After a 2 h adsorption period, cells were washed with PBS and incubated in the corresponding medium supplemented with 3% fetal bovine serum. At specific time points after infection, the amount of extracellular (MEFs) or cell-associated (macrophages) infectious virus present in the cultures was determined as previously described [65] by a standard plaque assay on MEFs.

### NK cell degranulation assays

NK cell degranulation was evaluated using the CD107a mobilization assay. Cultures of peritoneal macrophages, either mock-infected or infected with 10 PFU/cell of MCMV or MCMVΔm154 for 72 h, were incubated for 5 h at 37°C with purified NK cells in the presence of monensin (BD Biosciences) and anti-CD107a-Alexa Fluor 488, at an effector-to-target cell ratio (E/T) of 1∶1. NK cells treated with 0.5 µg/ml ionomycin (Sigma Aldrich) and 50 ng/ml PMA (Sigma Aldrich) were used as a positive control for degranulation. Cells were then washed in PBS supplemented with 2 mM EDTA, stained for 30 minutes at 4°C with anti-CD49b-PE/Cy7, recognizing DX5, and analyzed by flow cytometry. When stated, MCMVΔm154-infected macrophages were pre-incubated with 10 µg/ml of the indicated Fc fusion protein for 30 min at 37°C, cultures washed, and subjected to the CD107a mobilization assay using an E/T ratio of 0.5∶1.

### Mouse infections

Seven-week-old BALB/c.ByJ female mice were obtained from Harlan (Netherlands) and housed in the vivarium (University of Barcelona) under specific-pathogen-free conditions. Mice were i.p. inoculated with 5×10^5^ or 2×10^6^ PFU of tissue culture-propagated wt MCMV, MCMVΔm154, or MCMVΔm154Int recombinants. When specified, NK cells were depleted by i.p. injection of rabbit antiserum to asialo GM1 (Wako Pure Chemical Industries, Osaka, Japan) at a concentration of 25 µg per mouse, one day before infection and on day 2 after infection. The efficacy of depletion was assessed by cytofluorometric analyses of spleen cells using an anti-mouse pan-NK cell mAb CD49b-PE/Cy7. At designated times after infection, mice were sacrificed, and specific organs were removed and harvested as a 10% (weight/volume) tissue homogenate. Tissue homogenates were sonicated and centrifuged, and viral titers from the supernatants were determined by standard plaque assays, including centrifugal enhancement of infectivity [62] on MEFs. In experiments evaluating the cellular influx to the peritoneal cavity, mice were sacrificed 2 days after infection and cells present in the peritoneal cavity harvested with 5 ml of PBS. Total number of cells were determined with a cell counter, and stained with a combination of mAbs CD11b-PE and Gr-1-APC or mAbs CD3-Alexa Fluor 647 and IgM-FITC to distinguish the different cellular subsets (macrophages [CD11b^+^ Gr-1^−^], neutrophils [CD11b^+^ Gr-1^+^], T lymphocytes [CD3^+^] or B lymphocytes [IgM^+^]). The number of peritoneal macrophages infected *in vivo* was assessed by IE1 staining of peritoneal lavage-derived macrophages of mice infected for 16 h with 2×10^6^ PFU.

### Sequence analysis

The signal peptide cleavage site and the transmembrane region were predicted by using the SignalP 4.1 (www.cbs.dtu.dk/services/SignalP/) and the TMHMM 2.0 (www.cbs.dtu.dk/services/TMHMM-2.0/) servers, respectively. The N-glycosylation and O-glycosylation motifs of the protein were identified by using the NetNGlyc 1.0 Server, (www.cbs.dtu.dk/services/NetNGlyc), and the NetOGlyc 4.0 Server (www.cbs.dtu.dk/services/NetOGlyc/), respectively.

### Statistical analyses

Analyses were performed with GraphPad Prism software (version 3.03, GraphPad Software, San Diego, CA). Statistical significance of viral titers between experimental groups was determined with the Mann-Whitney test (two-tailed). *P*-values less or equal to 0.05 (*), 0.01 (**), and 0.001 (***) were considered statistically significant.

## Supporting Information

Figure S1 **Similar composition of the cellular influx into the peritoneal cavity of wt MCMV- and MCMVΔm154-infected mice.** Groups of 7-weeks-old BALB/c.ByJ female mice were i.p. inoculated with 2×10^6^ PFU of wt MCMV or MCMVΔm154, or left uninfected. Flow cytometry analysis was performed on cells extracted from the peritoneal cavity two days after infection of mice by staining with a combination of mAbs CD11b-PE and Gr-1-APC, or mAbs CD3-Alexa Fluor 647 and IgM-FITC. (A) The total cell number and the percentage of neutrophils (CD11b^+^ Gr-1^+^), macrophages (CD11b^+^ Gr-1^−^), T lymphocytes (CD3^+^), and B lymphocytes (IgM^+^) are indicated. Data shown are the mean values (± SEM) of three individual mice. Differences in values between the wt MCMV and the MCMVΔm154-infected groups are not statistically significant (p>0.05). (B) Results of the double staining for CD11b-PE and Gr-1-APC of one representative animal from each group are shown.
(TIF)
