# Correction: Cytomegalovirus m154 Hinders CD48 Cell-Surface Expression and Promotes Viral Escape from Host Natural Killer Cell Control

**DOI:** 10.1371/journal.ppat.1004121

**Published:** 2014-04-15

**Authors:** The PLOS Pathogens Staff

In the Funding section, a grant from REIPI is incorrectly omitted. The Funding statement should read: “This study was supported by the Ministerio de Ciencia e Innovación (MICINN, Spain) through grants SAF 2011-25155 (to AA), and SAF 2012-39536 (to PE), the REIPI grant RD12/ 0015/0018 (to AA and PE), and the TransMedRi grant, No. 256686 (FP7-REGPOT-2010-5; to SJ). NPC and DF were supported by a Formación de Profesorado Universitario fellowship and a Juan de la Cierva postdoctoral contract from the MICINN. The funders had no role in study design, data collection and analysis, decision to publish, or preparation of the manuscript.”
