# Supplementary figures and images for: Cytomegalovirus m154 Hinders CD48 Cell-Surface Expression and Promotes Viral Escape from Host Natural Killer Cell Control
Source: PLoS Pathog. 2014 Mar 13;10(3):e1004000. doi: 10.1371/journal.ppat.1004000 (PMC3953435; doi:10.1371/journal.ppat.1004000)

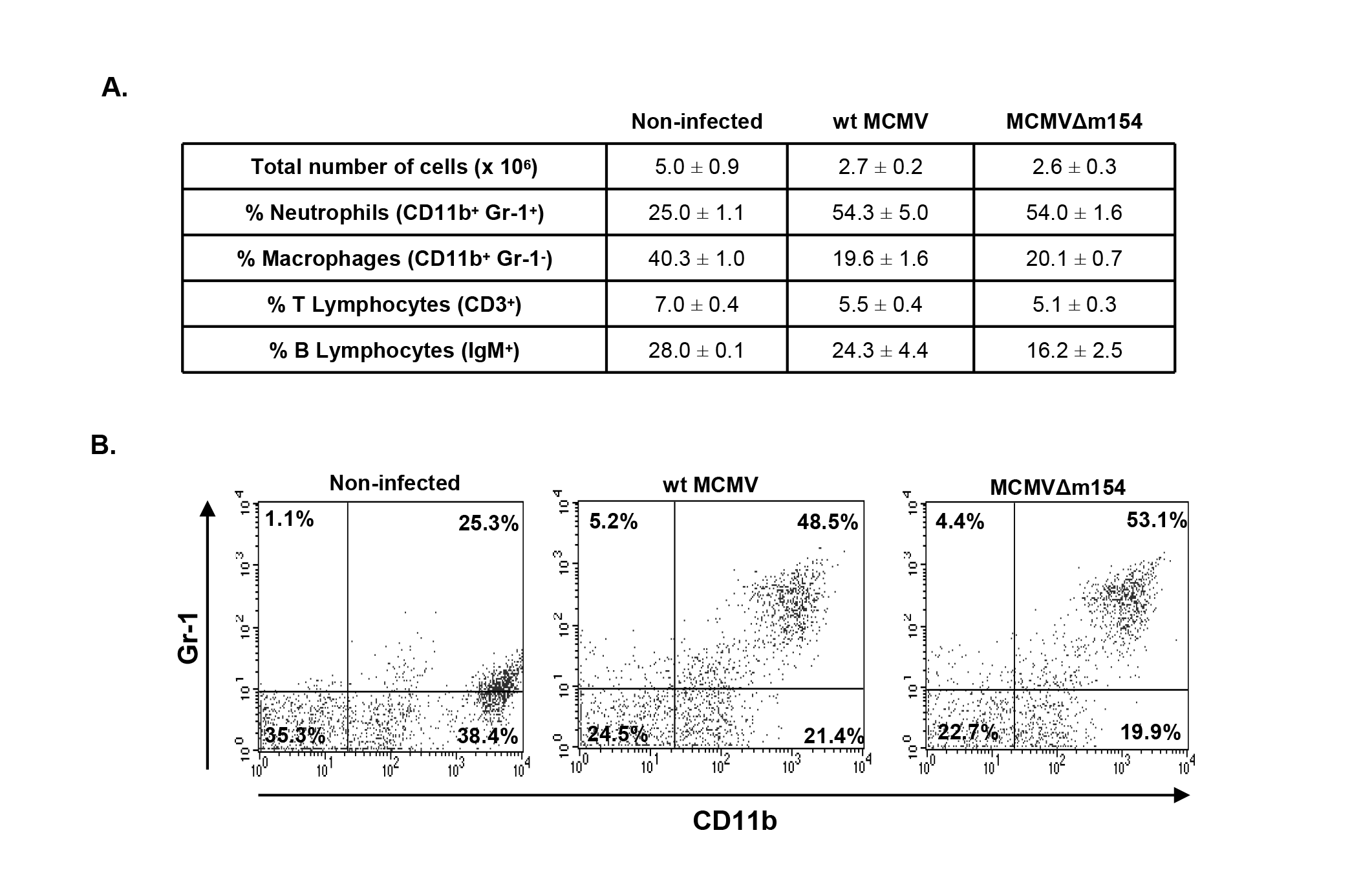

Supplement: Figure S1 — Similar composition of the cellular influx into the peritoneal cavity of wt MCMV- and MCMVΔm154-infected mice. Groups of 7-weeks-old BALB/c.ByJ female mice were i.p. inoculated with 2×10^6^ PFU of wt MCMV or MCMVΔm154, or left uninfected. Flow cytometry analysis was performed on cells extracted from the peritoneal cavity two days after infection of mice by staining with a combination of mAbs CD11b-PE and Gr-1-APC, or mAbs CD3-Alexa Fluor 647 and IgM-FITC. (A) The total cell number and the percentage of neutrophils (CD11b+ Gr-1+), macrophages (CD11b+ Gr-1−), T lymphocytes (CD3+), and B lymphocytes (IgM+) are indicated. Data shown are the mean values (± SEM) of three individual mice. Differences in values between the wt MCMV and the MCMVΔm154-infected groups are not statistically significant (p>0.05). (B) Results of the double staining for CD11b-PE and Gr-1-APC of one representative animal from each group are shown. (TIF) [file ppat.1004000.s001.tif]
